# The amoral atheist? A cross-national examination of cultural, motivational, and cognitive antecedents of disbelief, and their implications for morality

**DOI:** 10.1371/journal.pone.0246593

**Published:** 2021-02-24

**Authors:** Tomas Ståhl

**Affiliations:** Department of Psychology, University of Illinois at Chicago, Chicago, Illinois, United States of America; Middlesex University, UNITED KINGDOM

## Abstract

There is a widespread cross-cultural stereotype suggesting that atheists are untrustworthy and lack a moral compass. Is there any truth to this notion? Building on theory about the cultural, (de)motivational, and cognitive antecedents of disbelief, the present research investigated whether there are reliable similarities as well as differences between believers and disbelievers in the moral values and principles they endorse. Four studies examined how religious disbelief (vs. belief) relates to endorsement of various moral values and principles in a predominately religious (vs. irreligious) country (the U.S. vs. Sweden). Two U.S. M-Turk studies (Studies 1A and 1B, *N* = 429) and two large cross-national studies (Studies 2–3, *N* = 4,193), consistently show that disbelievers (vs. believers) are less inclined to endorse moral values that serve group cohesion (the binding moral foundations). By contrast, only minor differences between believers and disbelievers were found in endorsement of other moral values (individualizing moral foundations, epistemic rationality). It is also demonstrated that presumed cultural and demotivational antecedents of disbelief (limited exposure to credibility-enhancing displays, low existential threat) are associated with disbelief. Furthermore, these factors are associated with weaker endorsement of the binding moral foundations in both countries (Study 2). Most of these findings were replicated in Study 3, and results also show that disbelievers (vs. believers) have a more consequentialist view of morality in both countries. A consequentialist view of morality was also associated with another presumed antecedent of disbelief—analytic cognitive style.

## Introduction

Over the last 40 years, we have seen dramatic improvements in (explicit) attitudes toward most racial and religious minorities in the U.S. Improvements in attitudes toward atheists, however, appear to be lagging behind [1]. According to a Pew poll from 2019, Americans have a more unfavorable attitude toward atheists (and Muslims) than toward any other religious group [2]. Furthermore, whereas 93 to 96 percent of Americans reported being willing to vote for a qualified African American, Catholic, or Jewish presidential candidate in 2019, only 60 percent were willing to vote for a qualified atheist candidate [3].

Psychologists have recently started to examine *why* people have such antipathy toward atheists, and the findings strongly suggest that prejudice against atheists is driven by distrust [4–8]. According to a Pew poll from 2019, 44% of Americans think that belief in God is necessary for morality [9]. Distrust of atheists appears to stem from two related yet distinct concerns. First, people worry that atheists do not share their moral norms and values. In fact, atheists form the social group that Americans believe to be least in agreement with their vision of America [1]. Second, even if atheists were to have a general sense of right and wrong, people still worry that they would engage in immoral behavior because they do not believe in a monitoring God [7]. Notably, these concerns are not restricted to Americans or even to religious people. A recent study found strong evidence for the negative stereotype about atheists as immoral in 11 out of 13 countries investigated, and this stereotype was subscribed to even by atheists themselves [8].

Is there any validity to these widespread concerns about atheists having different moral standards than theists? The present research addresses this question by examining the extent to which disbelievers consider various values and principles relevant for morality, and to what extent their moral values and principles are similar to or different from those of religious believers. Results from a total of four studies are reported, including two large cross-national studies, comparing data from a country where religious belief is the norm (the U.S.), to data from one of the most secular countries in the world (Sweden). The central message in this article is that disbelievers *do* have a moral compass, but that it is calibrated somewhat differently than the one used by religious believers. Furthermore, suggestive evidence is provided, indicating that differences between disbelievers and believers regarding the moral values and principles they endorse may be attributable to the cultural, (de)motivational, and cognitive antecedents of their (dis)belief.

### Does religious belief promote morality?

Researchers have primarily addressed whether religious belief promotes morality by investigating the relationship between religiosity on the one hand, and the inclination to engage in various behaviors that are considered moral or immoral on the other. The results of this enterprise have been mixed. Although religiosity seems to be positively related to some morally relevant behaviors, it appears to be unrelated or negatively related to others [10–15]. Furthermore, acting in a way that can be considered moral does not imply that the behavior was morally *motivated*. Various other considerations, such as reputational concerns and ingroup favoritism, may also explain observed differences in morally relevant behavior [16, 17]. Similarly, acting in a way that can be considered immoral does not rule out that the behavior was morally motivated. History is full of examples of seemingly highly immoral acts that appear to have been driven by moral motives. For example, the 9/11 attacks, and the Oklahoma City bombing, seemed to be driven by strong moral convictions. In fact, some scholars have argued that most human violence is driven by moral motives [18]. An exhaustive review of the literature on the links between religiosity and moral behavior is far beyond the scope of this article. Instead, to illustrate the points raised above, I focus on two relevant areas of research—charity donations and prejudice—that suggest opposite relationships between religiosity and moral behavior.

In an extensive review of the literature on charity donations [11], Bekkers and Wiepking concluded that there are positive relationships between factors such as church attendance and religiosity on the one hand, and donations to charity on the other. However, they also noted that controlled experimental studies where donations are given in private rather than in public, and to secular rather than to specific religious organizations, have generally found no relationship between religiosity and charitable donations. Bekkers and Wiepking’s review suggests that the relationship between religiosity and charitable donations is likely to be explained to a considerable degree by factors such as the frequency with which one is solicited to donate (church attendants are more frequently solicited [19]), whether solicitation occurs in public [11], and whether the charity is associated with one’s ingroup (e.g., religious organization). Notably, religiosity has been linked to both ingroup favoritism [20–22] and impression management [23]. Thus, rather than implying that irreligious people are less morally sensitive to the plight of those in need, their weaker inclination to donate to charities may be due to various other factors, such as weaker reputational concerns, less ingroup favoritism, or less exposure to donation requests [24, 25].

A second morally relevant domain for which the role of religiosity has been examined is that of prejudice and discrimination toward minorities and stigmatized groups. Research in this area suggests that religiosity is associated with prejudice toward different minority groups. Specifically, religiosity is positively related to antipathy toward atheists [7, 8], racial minorities [12], and homosexuals [14]. One possible explanation of these findings is that religious people rely less on moral considerations than the nonreligious when they evaluate these minority groups. Alternatively, the religious may simply rely on *different* moral considerations than the nonreligious, and specific minority groups may be perceived as threats to religious’ people’s moral values, but not to the moral values of the nonreligious. For example, homosexuals may be perceived as a threat to traditional moral values about marriage and sex [26], whereas atheists may be perceived as a threat to the religious moral community more generally [1, 7].

As the examples above illustrate, the relationships between religiosity and morally relevant attitudes and behaviors can go in either direction, depending on the specific morally relevant attitude or behavior under examination. Furthermore, seemingly morally relevant attitudes and behaviors can be driven by a whole range of moral as well as amoral considerations. It therefore seems that a more fruitful way to start investigating the relationship between religious disbelief and morality is to examine the similarities and differences between believers and disbelievers in the extent to which they view various *values* and *principles* as relevant and important for morality. To the extent that differences in moral values and principles between believers and disbelievers are found, researchers can then turn to investigate their behavioral consequences, along with the consequences of various amoral considerations.

The present research investigates whether disbelievers differ from believers in how they conceptualize morality. An underlying assumption is that, to the extent that disbelievers’ views of morality are different from those of believers, these differences are unlikely to be attributable to religious disbelief itself. Rather, any unique aspects of disbelievers’ views of morality are expected to be attributable to the cultural, (de)motivational, and cognitive factors that *caused* their disbelief. I shall therefore begin by discussing recent insights about the antecedents of religious disbelief. These insights are then integrated with current theorizing in moral psychology to generate a set of hypotheses about how moral values and principles may differ between disbelievers and believers.

### Antecedents of religious disbelief

Cognitive scientists have proposed four different processes through which people can become religious disbelievers [27]. First, religious beliefs are thought to be powerfully culturally transmitted through so-called credibility-enhancing displays (CREDs). Children are more likely to accept certain religious beliefs as true to the extent that they observe important others in their community engage in behaviors in the service of those beliefs, that would be personally costly if the beliefs were false (e.g., attend religious meetings, perform demanding religious rituals, engage in pro-social behavior). By contrast, to the extent that such CREDs are absent in the community while growing up, children are far less likely to adopt any religious beliefs even if their parents are religious [28, 29]. Thus, an absence of CREDs in the community is thought to constitute a path to religious disbelief.

Second, scholars have long argued that belief in a powerful deity can provide a sense of compensatory control and comfort during times of suffering and existential uncertainty [27]. Experimental studies support this notion, as manipulations of suffering [30], mortality salience [31, 32], loneliness [33], and loss of control [34, 35], each strengthen religious belief. Moreover, religious belief is stronger in countries associated with high levels of child mortality, poverty, and various indicators of societal instability [30, 36]. By contrast, religious disbelief is most prevalent in the Scandinavian countries, the safest and most stable region in the world [36, 37]. Thus, growing up in an environment where there are low levels of (perceived) existential threat/uncertainty should reduce the motivation to believe in powerful deities, and thereby provide a possible path to disbelief.

Third, people can reason themselves into religious disbelief [27]. To the extent that religious belief is reliant on various intuitive (System 1) social cognitive processes, an inclination to over-ride such processes with more analytic (System 2) thinking could reduce religious belief. Consistent with this line of reasoning, multiple labs have documented a reliable negative relationship between analytic cognitive style (ACS) and religious belief in North America [38–40]. A recent meta-analysis (*k* = 31, *N* > 15,000) found that this relationship is reliable (albeit weak [41]). However, the relationship between ACS and religious disbelief appears to primarily hold up in WEIRD (Western, Educated, Industrialized, Rich, Democratic) countries [42]. One possible explanation for this finding is that the relationship between ACS and religious disbelief may depend on the strength of cultural norms to believe in God. For example, the relationship between ACS and religious disbelief may fail to emerge in cultures where norms to believe in God are very strong [43]. Alternatively, the relationship between ACS and religious disbelief may be curvilinear. That is, the relationship may disappear when cultural norms to believe in God are either very strong, or when there are no cultural norms to believe in God (and thus no intuitive beliefs for analytic thinking to override).

Finally, it has also been proposed that belief in a deity may rely on mentalizing abilities [27]. According to this line of thought, people who struggle to understand other people’s intentions and mental states (e.g., individuals on the autism spectrum), should also have a hard time conceptualizing an invisible, supernatural agent. As a consequence, individuals with low mentalizing abilities may be less inclined to believe in God. To date, however, the evidence in support for the relationship between mentalizing abilities and religious belief is weak. Although some studies have found a link between mentalizing abilities and belief in a personal God [44], a recent set of very large cross-national studies (*N* > 67,000) failed to find any relationships between religious belief and several validated measures of mentalizing abilities [29]. Thus, it seems implausible that mentalizing deficits serve as a reliable antecedent of religious disbelief.

In summary, there appear to be at least three plausible contributors to religious disbelief: a lack of exposure to CREDs in the community while growing up, living under conditions of low existential threat, and a reliance on an analytic (vs. intuitive) cognitive style should all increase the likelihood of becoming a disbeliever. In the following sections I connect these insights to the literature on moral psychology, and postulate a set of tentative hypotheses about how each of these factors may contribute to disbelievers’ views on morality.

### Antecedents of disbelief and the moral foundations

How do the cultural, (de)motivational, and cognitive antecedents of disbelief reviewed above shape people’s views about morality? To address this question, I rely on the conceptualization of the moral domain provided by Moral Foundations Theory [45, 46]. Although this theory is not without its critics [47–49] it is arguably the most ambitious, and most frequently used taxonomy of human moral values. It is therefore a suitable theoretical framework for the purposes of the present research. According to MFT, the human moral domain consists of (at least) five foundations: Care/harm, Fairness/cheating, Loyalty/betrayal, Authority/subversion, and Sanctity/degradation. All of the moral foundations are thought to be the result of evolutionary processes, yet their significance as well as their manifestations are viewed as highly responsive to cultural influence. Thus, societies as well as individuals are expected to differ in the extent to which they view each of the foundations as relevant for morality, as well as in how those foundations manifest themselves in specific moral attitudes and behavior. The Care/harm and Fairness/cheating foundations are thought to serve to protect the well-being of vulnerable individuals, and are therefore referred to as *individualizing moral foundations*. By contrast, the proposed main function of Loyalty/betrayal, Authority/subversion, and Sanctity/degradation is to protect the integrity of the group, and these foundations are therefore conceptualized as *binding moral foundations*.

Graham and Haidt have argued that a core function of religion is to bind group members together into cohesive communities [21]. Based on this line of reasoning, believers (vs. disbelievers) should be more inclined to endorse moral values that serve group cohesion (the binding moral foundations). Consistent with this analysis, there is some evidence that religious commitment is positively associated with endorsement of all of the binding foundations, and that these associations remain when controlling for political orientation [50]. But how do these values spread through the religious community? One possible answer to that question is that the binding moral foundations may transfer to younger generations through exposure to various CREDs that signal the fundamental importance ascribed to the religious community. As stated above, religious CREDs are behaviors performed in the service of specific religious beliefs, that would be personally costly if the beliefs were false. CREDs thereby signal the importance and credibility of those beliefs. Notably, however, many CREDs simultaneously signal the individual’s commitment to the community (donations, volunteer work), and to the cohesiveness of that community (attending religious meetings, public religious rituals, engaging in religious chants). It therefore seems plausible that CREDs not only increase the credibility of specific religious beliefs, but also the perceived *moral significance* of the community and the binding moral foundations themselves. By contrast, to the extent that CREDs are absent in the community in which people grow up, people should be less inclined to believe in God—and may also be less inclined to endorse the binding moral foundations.

There is also evidence that various existential threats promote group cohesion and ingroup favoritism, and might therefore also contribute to the moral significance ascribed to the binding moral foundations. For example, manipulations of mortality salience increase ingroup favoritism [51] and nationalistic bias [52]. In a similar vein, threats to one’s need to belong [53], and one’s sense of personal control [34], increase conformity and support for the national government, respectively. Finally, there is some direct evidence that individual differences in belief in a dangerous world predict endorsement of the binding moral foundations [54]. Based on this literature, low (vs. high) levels of existential threat should make people less inclined to believe in God—and less inclined to endorse the binding moral foundations.

There is also some empirical evidence that individual differences in ACS contribute to the perceived significance of the binding moral foundations. Specifically, individuals with a more analytic (vs. intuitive) cognitive style have been found to be somewhat less inclined to endorse the binding moral foundations, and to view disgusting (but harmless) acts as less immoral [55, 56]. Based on these initial findings, individuals who score high (vs. low) on ACS should be less inclined to believe in God—and less inclined to endorse the binding moral foundations.

Finally, mentalizing abilities are closely associated with the ability to empathize with and be concerned about victims of misfortune. Higher (vs. lower) mentalizing abilities should therefore be associated with stronger endorsement of the moral foundations that center on the well-being on individuals (the individualizing moral foundations). However, as stated earlier, the bulk of the evidence suggests that mentalizing abilities are unrelated to religious belief. Thus, although low (vs. high) mentalizing abilities should be associated with weaker endorsement of the individualizing moral foundations, it seems implausible that individual differences in mentalizing abilities should produce differences in endorsement of the individualizing moral foundations between believers and disbelievers.

To summarize, there are theoretical and empirical reasons to expect that a relative absence of CREDs, low levels of existential threat in the community, as well as high ACS should all serve to constrain the scope of the moral domain by attenuating the moral significance ascribed to the binding moral foundations. Because lower levels of CREDs, lower existential threat, and higher ACS are expected to promote disbelief, there are also reasons to suspect that these factors may contribute to weaker endorsement of the binding moral foundations among disbelievers (vs. believers). In addition, lower mentalizing abilities should constrain the moral domain by reducing the moral significance ascribed to the well-being of vulnerable individuals (the individualizing foundations). However, because the empirical evidence speaks against a link between mentalizing abilities and religious belief, there is no reason to expect any differences in endorsement of the individualizing moral foundations between believers and disbelievers. In the following section I go beyond the moral foundations and discuss how antecendents of disbelief may contribute to differences between believers and disbelievers in the processes through which they make moral judgments, as well as in the extent to which they view epistemic rationality as a moral issue.

### Beyond the moral foundations: Consequentialist thinking and moralized rationality

Multiple studies suggest that religious disbelief is associated with a more consequentialist view of morality. Specifically, disbelievers are more inclined to judge the appropriateness of actions based on their consequences relative to the consequences of inaction [57–59]. There are reasons to suspect that this relationship may be attributable to differences in ACS, because a reliance on more analytic (System 2) cognitive processes is associated with a tendency to make consequentialist moral judgments [57, 60–63]. Based on this line of reasoning, individuals who score high (vs. low) on ACS should not only be less inclined to believe in God, but also be more inclined to endorse a consequentialist view of morality.

Finally, religious disbelief has also been linked to moralization of epistemic rationality itself. Specifically, weaker religious belief is associated with viewing reliance on logic and evidence when forming and evaluating beliefs as a moral good [64]. Thus far, no studies have systematically examined where the association between religious disbelief and moralizing epistemic rationality may come from. One possibility is that this relationship is attributable to differences in ACS between believers and disbelievers. To the extent that one is inclined to rely on analytic thinking, one may also be more inclined to view this cognitive style as morally superior to alternative ways of forming and evaluating beliefs. However, the only two studies that included data on both ACS and moralized rationality found no support for that prediction [65]. This possibility will be examined more rigorously in the present research.

In summary, there are theoretical as well as empirical reasons to expect disbelievers to be less inclined than believers to endorse the binding moral foundations, and we propose that this difference may be attributable to varying levels of exposure to CREDs, existential threat, as well as to individual differences in cognitive style. Furthermore, previous research suggests that disbelievers (vs. believers) should be more inclined to endorse a consequentialist (vs. deontological) view of morality, as well as to view epistemic rationality as an important moral value. These two differences between disbelievers and believers may also be attributable to individual differences in cognitive style.

### Overview of studies

Four online surveys were conducted to examine the relationships between religious disbelief (vs. belief) and views about morality. First, American M-Turk workers were recruited to explore how belief strength relates to endorsement of the moral foundations (Study 1A). This initial study was directly replicated, while controlling for socially desirable responding (Study 1B). The link between disbelief (vs. belief) and the moral foundations was further examined in Study 2, this time using a much larger U.S. sample, as well as an equally large sample from a predominantly non-religious country (Sweden). The analysis was further expanded by exploring differences in the inclination to moralize epistemic rationality, and by examining how exposure to CREDs and existential threat relate to moral value endorsement. Finally, another large cross-national sample (U.S. and Sweden) was collected in Study 3 to investigate how individual differences in ACS, mentalizing abilities, and exposure to CREDs relate to endorsement of the moral foundations, moralization of epistemic rationality, and to consequentialist (vs. deontological) moral reasoning.

## Studies 1A & 1B

The purpose of Study 1A was to provide a first test of the prediction that disbelievers (vs. believers) are less inclined to endorse the binding moral foundations. To that end, measures of religiosity, and endorsement of the five moral foundations were administered. Because religiosity is associated with political conservatism, which in turn is associated with stronger endorsement of the binding moral foundations [45], political orientation was also assessed. For exploratory purposes, a measure of endorsement of Liberty/oppression—which has been put forth as a possible sixth moral foundation [66]—as well as a measure of amoral tendencies were also included. The main purpose of Study 1B was to replicate the results from Study 1A, while controlling for differences in socially desirable responding. This was an important step, because previous research suggests that religiosity is positively associated with socially desirable responding [23]. With the exception of adding a measure of socially desirable responding, Study 1B was an exact replication of Study 1A.

## Method

### Participants

These studies were approved by the Office for the Protection of Research Subjects (OPRS) at the University of Illinois at Chicago (Protocol:2014–0220). Participants in both studies were recruited online from Amazon’s Mechanical Turk. They received $2.50 for their participation in Study 1A or 1B, as well as a subsequent study that was not relevant for the present research. Participation lasted approximately 30 minutes in total.

#### Study 1A

This sample consisted of 179 MTurk workers residing in the US (81 Christians, 69 “nones” [36 atheists, 29 agnostics, 4 other], 4 Muslims, 2 Jews, 23 other). Fifty-five percent of participants were male (*M*_age_ = 34.22, *SD* = 11.99). One hundred thirty-five were Caucasian, 27 Asian American, 7 African American, 6 Hispanic/Latino, and 2 other.

#### Study 1B

This sample consisted of 250 MTurk workers residing in the US (117 Christians, 110 “nones” [48 atheists, 45 agnostics, 17 other], 5 Muslims, 4 Jews, 14 other). Fifty-one percent of the sample were male (*M*_age_ = 32.87, *SD* = 10.57). One hundred ninety-one were Caucasian, 22 Asian American, 16 African American, 16 Hispanic/Latino, 4 Native American, and 1 other.

### Procedure and materials

Upon ensuring that they were 18 years or older and giving informed consent, participants filled out the survey online. They were given one hour to complete the study. At the end of the study, all participants were thanked and paid for their participation.

#### Education

Level of education was measured on a 6-point scale: (1 = *Less than high school*, 2 = *High school*, 3 = *Some college*, *but no diploma*, 4 = *Bachelor’s degree*, 5 = *Master’s degree*, 6 = *Doctoral degree*).

#### Political orientation

Political leanings were assessed on a 7-point scale (1 = *very liberal*, 7 = *very conservative*)

#### Religiosity

Level of religiosity (vs. irreligiosity) was measured using a thermometer slider (0 = *Extremely nonreligious*, 100 = *Extremely religious*).

#### Moral foundations

Endorsement of the five moral foundations was measured using the MFQ30 [67]. There are six items for each moral foundation. Each item is measured using a 6-point scale, where higher numbers indicate stronger endorsement of the moral foundation. In all studies reported in this article, scores on Care/harm and Fairness/cheating were collapsed to create a reliable measure for the individualizing moral foundations, and scores on Authority/subversion, Loyalty/betrayal, and Sanctity/degradation were collapsed to create a reliable measure for the binding moral foundations. For all studies, I have also conducted alternative analyses looking at each moral foundation separately. These analyses produce highly consistent results, and are presented in Tables M-R in S1 Text.

#### Liberty/oppression

Endorsement of Liberty/oppression was measured using the 9-item scale developed by Iyer and colleagues [66]. This scale contains items such as: “People should be free to decide what group norms or traditions they themselves want to follow,” and “Society works best when it lets individuals take responsibility for their own lives without telling them what to do.” As with the MFQ30, items are measured on a 6-point scale, where higher numbers indicate stronger endorsement of Liberty.

#### Amorality

The five-item amorality subscale from the Machiavellian Personality Scale was used to measure amoral tendencies [68]. This scale contains items such as: “I am willing to be unethical if I believe it will help me succeed.” All items were answered on a 5-point scale (1 = *Not at all*, 5 = *Very much*).

#### Balanced Inventory of Desirable Responding (BIDR, Study 1B)

The one addition to Study 1B was the BIDR [69]. This scale was presented after the measures of religious affiliation and religiosity, but before all of the morally relevant scales. The BIDR consists of two subscales, measuring different aspects of desirable responding: self-deceptive enhancement (SDE) and impression management (IM). Whereas SDE is a measure of desirable responding resulting from an inflated self-image, IM measures more strategic attempts to present oneself in an overly positive light to others. The BIDR consists of 40 items, 20 of which measure SDE, and 20 of which measure IM. For each item, participants are asked to indicate on a 7-point scale the extent to which a statement is true or not true of them (1 = *Not true*, 7 = *Very true*). An example item from the SDE subscale is: “I don’t care to know what other people really think of me.” An extreme response on this item (i.e., 6 or 7) generates 1 point on the SDE subscale, whereas a response lower than 6 is scored as 0. On reversed items, a response of 1 or 2 generates 1 point on the SDE subscale, whereas a response higher than 2 is scored as 0. Thus, scores can range from 0 to 20 on the SDE subscale. An example item from the IM subscale is “When I hear people talking privately, I avoid listening.” Scoring is done the same way for the IM subscale as for the SDE subscale.

## Results

Means, standard deviations, and reliability statistics for all scales, as well as zero-order correlations between all variables are presented in Tables A and B in S1 Text. The relationships between religiosity and the morally relevant variables were examined using hierarchical regression analyses. Gender, age, and level of education were always entered in Step 1. In analyses for Study 1A, the measure of religiosity was entered in Step 2. For Study 1B, IM and SDE were entered in Step 2, and religiosity in Step 3, in order to examine whether controlling for socially desirable responding affected relationships between religiosity and the morally relevant variables in any substantive way. Whenever a reliable relationship between religiosity and a morally relevant variable was obtained, another regression analysis was conducted, in which political orientation was added as an additional control variable.

### Study 1A

Results of the hierarchical regression analysis are summarized in Table C in S1 Text. As predicted, being more irreligious was associated with substantially weaker endorsement of the binding moral foundations (*R*^2^ = .32). Notably, this relationship remained when controlling for political orientation, although the amount of variance explained by religiosity was reduced substantially (*R*^2^ = .17). Religiosity was unrelated to endorsement of the individualizing moral foundations, Liberty/oppression, as well as to amoral tendencies.

### Study 1B

Results are summarized in Table D in S1 Text. The association between religiosity and endorsement of the binding moral foundations found in Study 1A was replicated, while controlling for individual differences in IM and SDE (*R*^2^ = .22). Excluding IM and SDE from the model slightly strengthened the relationship between religiosity and endorsement of the binding moral foundations (*R*^2^ = .26). Thus, although socially desirable responding inflates the association between religiosity and endorsement of the binding moral foundations to some extent, most of the relationship appears to be unrelated to social desirability concerns. Moreover, the relationship between religiosity and endorsement of the binding moral foundations remained when controlling for political orientation, although it was substantially weaker (*R*^2^ = .11). Unlike in Study 1A, religiosity was also positively associated with endorsement of the individualizing moral foundations(*R*^2^ = .01). However, this relationship disappeared completely when controlling for political orientation (*R*^2^ = .00). Finally, and consistent with results from Study 1A, religiosity was unrelated to endorsement of Liberty/oppression, as well as to amoral tendencies.

## Discussion

The results of Study 1A and Study 1B consistently showed that irreligiosity was strongly associated with weaker endorsement of the binding moral foundations, and that this relationship remained when controlling for differences in political orientation. By contrast, endorsement of the individualizing moral foundations was unrelated to irreligiosity in Study 1A, and only weakly (negatively) associated with irreligiosity in Study 1B. Moreover, the weak negative associatition between irreligiosity and endorsement of the individualizing moral foundations disappeared when controlling for differences in political orientation. The two studies also yielded consistent results regarding the relationships between religiosity, Liberty/oppression, and amoral tendencies. In both studies, religiosity was unrelated to endorsement of Liberty/oppression, as well as amoral tendencies. Taken together, these initial findings suggest that there is *one* substantial difference in conceptualizations of morality between disbelievers and believers: disbelievers (vs. believers) are less inclined to endorse the binding moral foundations.

Although suggestive, these studies have several limitations. First, data collection was limited to American M-Turk workers. It is not clear whether similar patterns would emerge in a broader segment of the U.S. population, much less in a different culture. To address this issue, large samples from professional survey companies were used in the subsequent studies, which enabled coverage of a much broader segment of the U.S. population, as well as to compare responses of Americans to responses from a population where being religious is not the norm (Swedes).

Second, belief (vs. disbelief) in God was assessed with a measure of religiosity in these initial studies. This is potentially problematic because religiosity is a much broader concept than belief (vs. disbelief) in God. The results obtained in Studies 1A and 1B may therefore be attributable to aspects of religiosity that have little to do with belief in God (e.g., sense of community, engagement in collective rituals). In subsequent studies I therefore focused specifically on people’s belief (vs. disbelief) in God. Finally, the analysis was also expanded by including measures of the inclination to moralize epistemic rationality, as well as various presumed antecedents of disbelief. Due to strict space limitations for these large-scale surveys, Study 2 focused exclusively on the role of cultural (CREDs) and (de)motivational (existential threat) antecedents of disbelief, whereas the role of presumed cognitive antecedents of disbelief were examined in Study 3. Thus, the goal of Study 2 was to examine (1) whether the association between belief (vs. disbelief) and the binding moral foundations held up in two large national samples with drastically different norms about religious belief, (2) whether this relationship can be attributed to differences in exposure to CREDs and existential threat, as well as (3) whether disbelief is associated with stronger endorsement of epistemic rationality as a moral value.

## Study 2

### Method

#### Participants

This study was approved by the Office for the Protection of Research Subjects (OPRS) at the University of Illinois at Chicago (Protocol:2017–0796). Participants were recruited from the online panel provided by Survey Sampling International. To try to ensure sufficiently large numbers of disbelievers and believers from both the U.S. and from Sweden, 500 individuals who reported a religious affiliation (vs. no religious affiliation) were requested from each country. Notably, however, being religiously affiliated (vs. unaffiliated) is not the same as being a religious believer (vs. disbeliever), and the religious affiliation quotas did not yield similar numbers of believers (vs. disbelievers) in each country. A total of 2,925 participants were recruited. Participants who reported not knowing whether they believed in God, or who failed to answer this question, were excluded (794 participants were excluded), resulting in a final sample of 2,131 participants. The final sample comprised of 810 believers and 278 disbelievers residing in the U.S., and 401 believers and 642 disbelievers residing in Sweden.

In the U.S. sample (*M*_age_ = 39.46, *SD* = 13.53), 38.2% were female, 31.9% male, 0.3% other, and 29.6% did not disclose their gender. In the Swedish sample, (*M*_age_ = 41.21, *SD* = 14.17), 31.5% were female, 32.9% male, 0.2% other, and 35.4% did not disclose their gender. In the U.S. sample, 62.8% were affiliated with Christianity, 30.4% were “nones” (17.4% Atheists, 2.3% Agnostics, 1.2% Secular Humanists, and 5.1% other), 2.5% Jewish, 0.7% Muslims, and 3.6% other. In the Swedish sample, 49.7% were affiliated with Christianity, 40.9% were “nones” (20.3% Atheists, 3.7% Agnostics, 3.3% Secular Humanists, and 3.7% other), 4.3% Muslims, 0.7% Jewish, 4.2% other. The U.S. sample was 52.6% Caucasian, 6.4% African American, 6% Hispanic/Latino, 3.4% Asian American, 0.8% Native American, 1.1% other, and 29.7% did not disclose their race/ethnicity. No data on race/ethnicity was collected in the Swedish sample, as it is not customary to do so in Sweden.

#### Procedure and materials

Upon giving informed consent, and ensuring that they were 18 years or older, participants were redirected to take part in the online survey.

#### Moral foundations

Exactly the same scales were used as in the previous studies.

#### Moralized rationality

The 9-item Moralized Rationality Scale [64] was used to measure to what extent people viewed epistemic rationality as an important moral value. This scale includes items such as: “Being skeptical about claims that are not backed up by evidence is a moral virtue”, and “It is immoral to hold irrational beliefs” (1 = *Completely disagree*, 7 = *Completely agree*).

#### CREDs

Exposure to credibility-enhancing displays in the community where people grew up was measured using a slightly modified version of the 7-item CREDs Exposure Scale [70]. The scale included items such as “Overall, to what extent did people in your community attend religious services or meetings?”, and “Overall, to what extent did people in your community make personal sacrifices to religion?” (1 = *to no extent at all*, 7 = *to an extreme extent*). Scores were averaged to create a reliable CREDs exposure scale.

#### Existential threat

The 12-item belief in a dangerous world scale (BDW) was used to measure existential threat [71, cf. 54]. This scale contains items such as “There are many dangerous people in our society who will attack someone out of pure meanness, for no reason at all”, and “Any day now, chaos and anarchy could erupt around us. All the signs are pointing to it” (1 = *Completely disagree*, 7 = *Completely agree*). Scores were averaged to create a reliable BDW scale.

#### Belief vs. disbelief

Participants were asked “Do you believe that there is a God?” (Yes, No, Don’t know). As indicated above, those who answered “Don’t know”, or failed to answer this question, were excluded from further analyses. The survey also included a measure of belief (vs. disbelief) strength. Participants who answered Yes (vs. No) to the question “Do you believe that there is a God” were redirected to the appropriate version of the follow-up question “How confident are you that there is a God (vs. is no God)?” (1 = *Not at all confident*, 7 = *Extremely confident*). Responses to the disbelief strength version of this question were re-coded (-1 = *Not at all confident*, -7 = *Extremely confident*), and then merged with responses to the belief strength version to create a 14-point belief strength scale (-7 = *Extremely confident that there is no God*, 7 = *Extremely confident that there is a God*). Analyses of the belief strength measure revealed that it provided little information beyond whether or not participants were believers (vs. disbelievers), as responses clustered around the two extremes of the scale. Because the measure deviated so drastically from normality, I decided to focus exclusively on the dichotomous belief (vs. disbelief) measure. Alternative analyses, using the continuous belief strength measure, are reported in S1 Text, and they produce almost identical results.

#### Political orientation

Political leanings were assessed with two items: “How would you describe your political outlook with regard to social issues?”, How would you describe your political outlook with regard to economic issues?” (1 = *very liberal*, 7 = *very conservative*). These two items were averaged to create a reliable political orientation scale (*r* = .80, p < .001).

#### Demographics

Gender, age, level of education, and religious affiliation, were assessed as in the previous studies.

## Results

### Predicting disbelief

Means, standard deviations, and reliability statistics for all scales, as well as zero-order correlations between all variables are presented in Table E in S1 Text. The relationships between the presumed antecedents of disbelief and self-reported disbelief were examined using a hierarchical logistic regression, with disbelief as our criterion (1 = Believe there is a God, 0 = Believe there is no God). Country of residence (1 = U.S., 0 = SWE) was entered in Step 1, and the presumed antecedents of disbelief (CREDs, BDW) were standardized and entered in Step 2. Interactions between country of residence and the presumed antecedents of disbelief were entered in Step 3. Because so many participants failed to fill out either their gender, age, or level of education in this study, analyses are reported without these controls to avoid drastic data loss. Analyses with these controls included are reported in S1 Text, and they produce almost identical results.

Results of the hierarchical logistic regression analysis are presented in Table F in S1 Text. Being Swedish (vs. American) was associated with a higher probability of being a disbeliever (*OR* = .21). Moreover, less exposure to CREDs was associated with a higher probability of being a disbeliever (*OR* = 2.92), as were lower levels of BDW (*OR* = 1.57). The country by BDW interaction was also significant, whereas the country by CREDs interaction was not. Although lower BDW scores were associated with a higher probability of being a disbeliever in both countries, this relationship was stronger in the U.S. (*b* = .76, SE = .09, χ^2^(1) = 69.27, *p* < .001, *OR* = 2.13), than in Sweden (*b* = .26, SE = .07, χ^2^(1) = 13.66, *p* < .001, *OR* = 1.29).

To conclude, more Swedes than Americans were disbelievers in this sample. More importantly, although causal conclusions cannot be drawn based on correlational data, the results obtained were consistent with the notion that low exposure to CREDs in the community, and lower levels of existential threat (BDW), are antecedents of disbelief. BDW was a somewhat stronger negative predictor of disbelief in the U.S. (vs. Sweden), whereas exposure to CREDs was an equally strong negative predictor of disbelief in both countries.

### Disbelief and moral values

Associations between disbelief and moral values were examined in the same way as in previous studies (for analyses with control variables, see S1 Text). As can be seen in Table G in S1 Text, Americans (vs. Swedes) were more inclined to endorse the binding moral foundations (*R*^2^ = .08), and Liberty/oppression (*R*^2^ = .04), and slightly more inclined to endorse the individualizing moral foundations(*R*^2^ = .01). By contrast, Swedes (vs. Americans) were very slightly more inclined to moralize epistemic rationality (*R*^2^ = .003). More importantly, and consistent with Studies 1A and 1B, disbelief (vs. belief) was associated with considerably weaker endorsement of the binding moral foundations (*R*^2^ = .125). Importantly, this association remained when controlling for political orientation, although the amount of variance explained was slightly reduced (*R*^2^ = .097). By contrast, disbelief accounted for less than 1% of the variance in individualizing moral foundations, Liberty/oppression, and moralization of epistemic rationality. Finally, the associations between disbelief and moral value endorsement did not differ substantially across cultures. Disbelief was a slightly stronger negative predictor of endorsement of the binding moral foundations, and Liberty/oppression, among Americans (vs. Swedes), but these interactions only accounted for 1.2% and 0.2% of the variance, respectively.

To summarize, the results from Study 1A and 1B were closely replicated in this Study. Disbelief (vs. belief) was associated with considerably weaker endorsement of the binding moral foundations in the U.S. Importantly, a similar, albeit somewhat weaker relationship between disbelief (vs. belief) and the binding moral foundations was found in the Swedish sample. This pattern of results strongly suggests that the association between religious belief and endorsement of the binding moral foundations cannot be explained by the normative status of religious belief. Moreover, as in Studies 1A-1B, this relationship remained while controlling for differences in political orientation. Disbelief was only very weakly related to endorsement of the individualizing moral foundations, as well as to Liberty/oppression, in both countries. Lastly, unlike what has been found in American M-Turk and Crowdflower samples [64, 65], disbelief was only very weakly related to moralization of epistemic rationality in both countries.

### Can theorized antecedents of disbelief explain why disbelievers are less inclined than believers to endorse the binding moral foundations?

Hierarchical regression analyses were conducted to address this question. Country of residence was entered in Step 1, followed by the antecedents of disbelief (CREDs, BDW) in Step 2. Disbelief was entered in Step 3, to examine its contributions to endorsement of the binding foundations once the variance explained by the antecedents of disbelief had been accounted for. Finally, the interaction terms between country of residence and the two presumed antecedents of disbelief were entered in Step 4.

As can be seen in Table H in S1 Text, both theorized antecedents of disbelief explained a large amount of variance in endorsement of binding moral foundations (*R*^2^ = .23). Less exposure to CREDs, and lower BDW were associated with considerably weaker endorsement of binding moral foundations. Moreover, once these associations had been accounted for, the negative association between disbelief and endorsement of binding moral foundations was weakened considerably (*R*^2^ = .029). Notably, these patterns were similar in both countries, as the country by CREDs and country by BDW interactions failed to account for any substantial amount of variance (*R*^2^ = .002). Taken together, these results are consistent with the notion that less exposure to CREDs and lower BDW help explain why disbelievers (vs. believers) are less inclined to endorse the binding moral foundations.

### Are cultural differences in endorsement of the binding moral foundations mediated by differences in exposure to CREDs and BDW?

A bootstrapped mediation analysis, using *Process* [72] (Model 4, 5,000 resamples, bias-corrected), was conducted to test this idea. The indirect effect of country of residence, through exposure to CREDs, on endorsement of the binding moral foundations, was significant, .350, *SE* = .03, 95% CI: (.304, .400), as was the indirect effect of country of residence through BDW, .047, SE = .01, 95% CI: (.024, .070). Once these indirect effects had been accounted for, the direct effect of country of residence on endorsement of the binding moral foundations was considerably weaker, .086, SE = .04, 95% CI: (.015, .157). Thus, the weaker endorsement of the binding moral foundations observed among Swedes (vs. Americans), can partially be accounted for by their lower exposure to CREDs, and lower scores on BDW.

## Discussion

The present study improved on Studies 1A and 1B in several important ways. Disbelief (vs. belief) was measured directly (rather than level of religiosity), data was collected from a predominantly religious, as well as a predominantly irreligious country, and considerably larger and more representative samples were used. With all of these methodological improvements, the main finding from Studies 1A and 1B was replicated, showing once again that religious disbelief is associated with considerably weaker endorsement of the binding moral foundations in both the U.S. and in Sweden. Moreover, disbelief was only very weakly related to endorsement of the individualizing foundations, and to Liberty/oppression in both countries. Disbelief was also only very weakly related to moralization of epistemic rationality in both countries. Notably, this finding is inconsistent with previous work that has relied on more restricted U.S. samples [64].

More importantly, the present findings were highly consistent with predictions about the antecedents of disbelief, and their contributions to disbelievers’ views about morality. A lack of exposure to CREDs, and low levels of existential threat (BDW), were closely related to religious disbelief. Moreover, these factors were strong predictors of endorsement of the binding moral foundations in both countries. As a consequence, once cultural differences in exposure to CREDs and BDW had been accounted for, Americans and Swedes were considerably more similar in their level of endorsement of the binding moral foundations.

Study 3 serves to examine the replicability of these findings. The analysis is also expanded by investigating the link between religious disbelief and consequentialist moral reasoning, as well as the role of two additional presumed antecedents of disbelief (ACS and mentalizing abilities). Unfortunately, adding these variables forced the exclusion of other measures to meet the strict time limitations on the survey. Because it was the least powerful predictor of disbelief in Study 2, the measure of existential threat (BDW) was removed from the survey, and an abbreviated version of the MFQ was used. To ensure identical numbers of believers and disbelievers in each sample, a different screener was used in Study 3. Rather than requesting 50% quotas based on religious affiliation, 50% quotas were set for people who indicated that they believe (vs. do not believe) that there is a God. People who answered “don’t know” to this question were excluded from participation.

## Study 3

### Method

#### Participants

This study was approved by the Office for the Protection of Research Subjects (OPRS) at the University of Illinois at Chicago (Protocol:2017–0796). Two thousand and sixty-two participants (1060 Americans, 1002 Swedes) were recruited from the online panel provided by OvationMR. To ensure that the sample exclusively consisted of (an equal number of) disbelievers and believers in both countries, participants were screened with the question: “Do you believe that there is a God?” (Yes, No, Don’t know). Answering “Don’t know” to this question led to exclusion from the study. This resulted in a total of 554 believers and 506 disbelievers in the U.S. sample, and 502 believers and 500 disbelievers in the Swedish sample. The U.S. sample was more female (54.2% vs. 36.2%), and older (*M* = 43.8, *SD* = 16.59 vs. *M* = 34.58, *SD* = 15.04) than the Swedish sample. In the U.S. sample, 45.8% were affiliated with Christianity, 39.8% were “nones” (16.7% atheists, 8.7% agnostics, 1.2 secular humanists, 5.2 other, 8% did not report their non-religious identity), 2.6% Jewish, 1.2% Muslims, 5.4% other, and 5.1% did not report their religious affiliation. In the Swedish sample, 47.7% were affiliated with Christianity, 36.1% were “nones” (19.2% atheists, 3% agnostics, 1.9% secular humanists, 3.4% other, 8.6% did not report their non-religious identity), 7.6% Muslims, 2.3% Jewish, 5.9% other, and 0.4% did not report their religious affiliation. The U.S. sample was 70.1% Caucasian, 8.1% African American, 6.3% Hispanic/Latino, 6% Asian American, 1.5% Native American, 2.7% other, and 5.3% did not report their race/ethnicity (data on race/ethnicity was not collected in the Swedish sample, as it is not customary to do so in Sweden).

#### Procedure and materials

Upon giving informed consent, ensuring that they were 18 years or older, and passing the screener (belief/disbelief), participants proceeded to take part in the online survey.

***Disbelief vs*. *belief*.** The screener question described above was used to measure disbelief vs. belief. The same measure of belief strength as in Study 2 was also included. Because scores were once again clustered around the extremes of this scale, I decided to focus on the dichotomous measure of belief (vs. disbelief) in our analyses. Alternative analyses using the continuous measure of belief strength yielded almost identical results, and are reported in S1 Text.

***Political orientation*.** We used the same two items as in Study 2 to assess political orientation, and they were combined to create a reliable scale (*r* = .77, *p* < .001).

***Analytic cognitive style*.** The original 3-item Cognitive Reflection Test (CRT) [73], as well as the 4-item CRT-2 [74] were used to measure Analytic cognitive style. However, item 3 from the CRT-2 was excluded, because it does not translate properly into Swedish (the Swedish names of the months of April and June are not common first names in Sweden). Correct answers to the six CRT items were added up to create a reliable ACS scale.

***Mentalizing*.** The short version of the Empathy Quotient (EQ-Short) was used to assess mentalizing abilities [75].

***CREDs*.** The same 7-item scale as in Study 2 was used to measure exposure to credibility-enhancing displays in the community [70].

***Moral foundations*.** Due to space limitations only the items from the first half of the MFQ30 were included, along with the equivalent questions from the Liberty/oppression scale. This resulted in a 3-item (rather than 6-item) measure for each of the five foundations, and a 2-item measure of Liberty/oppression. The abbreviated version of the Liberty/oppression scale had poor reliability (*r* = .33, p < .001).

***Moralized rationality*.** The MRS was once again used to measure the extent to which people moralize epistemic rationality [64].

***Consequentialist thinking*.** The 14-item Consequentialist Thinking Scale (CTS) was used to measure consequentialist versus deontological moral reasoning [59]. Each item asks the respondent about their position regarding a behavior (e.g., treason, killing, torture, abortion) that is typically or frequently viewed as immoral (1 = Never morally permissible” [deontological response], 2 = “Morally permissible if it will produce greater good than bad consequences” [weak consequentialist response], 3 = “Morally obligatory if it will produce greater good than bad consequences” [strong consequentialist response]). Scores were added up and averaged to create a reliable consequentialist thinking scale.

***Demographics*.** Gender, age, level of education, race/ethnicity (U.S. sample only), and political orientation were measured, as in previous studies.

Upon completing the survey, participants were thanked for their participation.

## Results

### Predicting disbelief

Means, standard deviations, and reliability statistics for all scales, as well as zero-order correlations between all variables are presented in Table I in S1 Text. The relationships between the presumed antecedents of disbelief and reported disbelief (vs. belief) were examined in the same way as in Study 2 (but with control variables), and results are presented in Table J in S1 Text. Less exposure to CREDs in the community was associated with a higher likelihood of being a disbeliever (*OR* = 2.78), as was a more analytic cognitive style (*OR* = .67). However, Mentalizing was not reliably related to disbelief. There was also a reliable interaction between ACS and country of residence. Although the relationship was robust in both countries, a more analytic cognitive style was associated with a higher likelihood of being a disbeliever among Swedes (*b* = -.509, SE = .078, χ^2^(1) = 42.53, *p* < .001, *OR* = .601), than among Americans (*b* = -.29, SE = .076, χ^2^(1) = 14.43, *p* < .001, *OR* = .748). No other relationships reached statistical significance.

Taken together, although conclusions about causality cannot be drawn, the results were highly consistent with the notion that low exposure to CREDs, and having an analytic cognitive style, are antecedents of disbelief. By contrast, and consistent with recent large-scale studies [29], no support was found for the notion that low mentalizing abilities serve as a reliable antecedent of disbelief.

### Disbelief and morality

The links between disbelief and moral values (and principles) were examined in the same way as in Study 2, with the exception that control variables were included in the equation. The results of these analyses are presented in Table K in S1 Text.

As in Study 2, Americans endorsed the binding foundations more than Swedes (*R*^2^ = .014). However, this cultural difference was considerably smaller than in Study 2, presumably because this sample contained comparable numbers of disbelievers and believers from each country. As in Study 2, Americans (vs. Swedes) also scored slightly higher on endorsement of individualizing moral foundations (*R*^2^ = .011), whereas Swedes scored very slightly higher than Americans on moralization of epistemic rationality (*R*^2^ = .002). Unlike in Study 2, there was no cultural difference at all in endorsement of Liberty/oppression. Finally, Swedes were slightly more inclined than Americans to endorse consequentialist thinking (*R*^2^ = .008).

More importantly, however, the relationships between disbelief and moral values were largely replicated in both countries. As in the previous studies, disbelief (vs. belief) was associated with considerably weaker endorsement of the binding moral foundations (*R*^2^ = .112). Furthermore, this relationship remained when controlling for differences in political orientation (*R*^2^ = .094). By contrast, disbelief (vs. belief) explained less than 1% of the variance in endorsement of the individualizing moral foundations, Liberty/oppression, and moralization of epistemic rationality. It is also worth noting that the cultural differences in associations between disbelief and these moral values were even smaller in this study, as all of the interaction effects explained less than 1% of the variance. Finally, disbelievers (vs. believers) were more inclined to endorse consequentialist thinking (*R*^2^ = .035), and this association remained when controlling for differences in political orientation (*R*^2^ = .031). The association between disbelief and consequentialist thinking was slightly stronger among Swedes than among Americans.

### Can theorized antecedents of disbelief explain differences in morality between believers and disbelievers?

Analyses were restricted to the binding moral foundations, and consequentialist thinking, as these moral considerations were the only ones that were predicted by disbelief (vs. belief). Analyses were conducted in the same way as in Study 2, with the exception that control variables were included in the equation., The results of these analyses are presented in Table L in S1 Text.

#### Binding moral foundations

Less exposure to CREDs, and a more analytic cognitive style were associated with weaker endorsement of the binding moral foundations. Lower mentalizing abilities were also associated with weaker endorsement of the binding moral foundations, and these three factors accounted for a considerable amount of variance (*R*^2^ = .265). Moreover, once the associations to these factors had been accounted for, disbelief only accounted for a very small amount of variance in endorsement of the binding moral foundations (*R*^2^ = .015), as did the interaction terms (*R*^2^ = .005). These results are consistent with the notion that the negative association between disbelief and endorsement of the binding moral foundations is predominantly due to underlying differences between believers and disbelievers in their level of exposure to CREDs, and in analytic cognitive style. Lower mentalizing abilities were also associated with weaker endorsement of the binding moral foundations. However, this association cannot account for differences in endorsement of the binding moral foundations between believers and disbelievers, as mentalizing abilities were unrelated to disbelief in this study.

#### Consequentialist thinking

A more analytic cognitive style, lower mentalizing abilities, and less exposure to CREDs were associated with more consequentialist thinking. It should be noted, however, that these variables only accounted for a modest amount of variance (*R*^2^ = .012). Furthermore, disbelief (vs. belief) remained a stronger predictor of consequentialist thinking when controlling for these factors (*R*^2^ = .03). These results suggest that, although the presumed antecedents of disbelief can account for a small portion of the association between disbelief and consequentialist thinking in both countries, there appear to be additional factors that contribute to more consequentialist thinking among disbelievers (vs. believers).

### Can cultural differences in endorsement of the binding moral foundations and consequentialist thinking be explained by differences in exposure to CREDs, ACS, and mentalizing abilities?

To examine this issue, a bootstrapped mediation analyses was conducted, using *Process* [72] (Model 4, 5,000 resamples, bias-corrected).

#### Binding moral foundations

Controlling for gender, age, and level of education, the indirect effect of country of residence, through exposure to CREDs, on endorsement of the binding moral foundations, was significant, .190, *SE* = .02, 95% CI: (.144, .237), as was the indirect effect of country of residence through ACS, .044, *SE* = .01, 95% CI: (.027, .066), and mentalizing, .039, *SE* = .01, 95% CI: (.019, .062). Moreover, once these indirect effects had been accounted for, the direct effect of country of residence on endorsement of the binding moral foundations was no longer significant, -.019, SE = .05, 95% CI: (-.108, .07). Thus, the stronger endorsement of the binding moral foundations observed among Americans (vs. Swedes), was fully accounted for by their higher exposure to CREDs, lower ACS, and higher Mentalizing abilities.

#### Consequentialist thinking

Controlling for gender, age, and level of education, the indirect effect of country of residence, through exposure to CREDs, on consequentialist thinking, was significant, -.008, *SE* = .004, 95% CI: (-.017, -.0009), as was the indirect effect of country of residence through ACS, -.005, *SE* = .002, 95% CI: (-.011, -.001), and mentalizing, -.006, *SE* = .002, 95% CI: (-.011, -.002). However, once these indirect effects had been accounted for, the direct effect of country of residence on consequentialist thinking was still significant, -.059, SE = .02, 95% CI: (-.096, -.023). Thus, the more consequentialist thinking observed among Swedes (vs. Americans), was only partially accounted for by their lower exposure to CREDs, higher ACS, and lower mentalizing abilities.

## Discussion

The results of this study closely replicated the findings from Study 2. Disbelievers (vs. believers) were once again less inclined to endorse the binding moral foundations in both countries, whereas disbelief explained miniscule amounts of variance in endorsement of the individualizing moral foundations, Liberty/oppression, and moralization of epistemic rationality. Also consistent with results of Study 2, exposure to CREDs was a strong predictor of endorsement of the binding moral foundations in both countries. Going beyond Study 2, higher levels of ACS were associated with lower levels of endorsement of the binding moral foundations. Moreover, once differences in exposure to CREDs and ACS had been controlled for, disbelief (vs. belief) explained only a small amount of variance in endorsement of the binding moral foundations. Thus, results were once again consistent with the notion that exposure to CREDs help explain why believers endorse the binding moral foundations more than disbelievers. This study also provided initial evidence consistent with the idea that differences in cognitive style help explain the negative association between disbelief and endorsement of the binding moral foundations. Results also showed that mentalizing abilities were associated with higher levels of endorsement of the binding moral foundations. Mentalizing abilities were also positively associated with stronger endorsement of the individualizing moral foundations. However, these relationships do not help account for differences in moral values between believers and disbelievers, as mentalizing abilities were unrelated to disbelief.

Disbelievers (vs. believers) were also more inclined to endorse consequentialist thinking, an association that was slightly stronger among Swedes (vs. Americans). Finally, there was some evidence consistent with the notion that differences in cognitive style help account for why disbelievers (vs. believers) are more inclined to endorse a consequentialist view of morality, as ACS was positively associated with consequentialist thinking, and also accounted for a small part of the difference in consequentialist thinking between disbelievers and believers. However, disbelief remained a stronger predictor of consequentialist thinking than ACS, mentalizing and CREDs. Thus, there appear to be additional unknown factors that contribute to higher levels of consequentialist thinking among disbelievers (vs. believers).

## General discussion

Although cross-cultural stereotypes suggest that atheists lack a moral compass [8], no studies to date have systematically examined to what extent disbelievers’ and believers’ conceptualizations of morality are distinct. The purpose of the present research was to fill that gap. Based on current theorizing in moral psychology, I identified a highly plausible difference in moral values between believers and disbelievers. Specifically, it has been proposed that a core function of religion is to create highly cohesive communities that serve to promote cooperation and to prevent free-riding as societies increase in size [21, 76, 77]. Based on Moral Foundations Theory, it has further been proposed that religious communities generally promote moral values that serve group cohesion [21]. Based on this analysis it was hypothesized that disbelievers (vs. believers) should be less inclined to endorse the binding moral foundations. Across four studies, strong support was found for this prediction. In two U.S. M-Turk samples (Studies 1A and 1B), as well as two large cross-national samples (Studies 2–3), disbelievers were considerably less inclined than believers to endorse the binding moral foundations. Notably, this was the case in a country where religious belief is the norm (the U.S.) as well as in one of the most secular countries in the world (Sweden). Thus, the negative association between disbelief and endorsement of the binding moral foundations was independent of the normative status of religious belief in society. Moreover, this association was only slightly weakened when controlling for socially desirable responding (Study 1B), and remained robust (albeit weaker) when controlling for political orientation (all four studies). By contrast, and again consistent across all studies, disbelief (vs. belief) only explained miniscule amounts of variance in endorsement of moral values that serve to protect vulnerable individuals (the individualizing moral foundations) and Liberty/oppression. Furthermore, disbelief (vs. belief) was unrelated to amorality (Studies 1A, 1B), and explained only a miniscule amount of variance in moralization of epistemic rationality (Studies 2–3).

The moral psychology literature also suggests that disbelievers are more inclined than believers to form moral judgments on the basis of the consequences of specific actions compared to inaction [57–59]. This finding was replicated in Study 3. American as well as Swedish disbelievers (vs. believers) were indeed more inclined to rely on consequentialist thinking, an association that was slightly stronger among Swedes. In short, the present four studies suggest that believers and disbelievers share similar moral concerns about protecting vulnerable individuals, Liberty/oppression, and about being epistemically rational. They also score equally low on amorality. However, disbelievers (vs. believers) are (1) considerably less inclined to endorse moral values that promote group cohesion, but (2) more inclined to form moral judgments about harm based on the specific consequences of actions.

### Explaining moral disagreements between believers and disbelievers

In addition to mapping the similarities and differences in the moral values and principles endorsed by disbelievers and believers, a second aim of the present research was to explore where moral psychological differences between these groups may stem from. A set of hypotheses were derived based on an integration of insights regarding cultural, (de)motivational and cognitive antecedents of disbelief [27] and current theorizing in moral psychology. First, many CREDs not only signal the credibility and importance of specific religious beliefs, but also of the broader religious community. Based on this observation it was proposed that lower exposure to CREDs may contribute to weaker endorsement of the binding moral foundations among disbelievers (vs. believers). Second, based on a substantial literature linking various existential threats to increased group cohesion [34, 51–54], it was proposed that lower existential threat may also contribute to weaker endorsement of the binding moral foundations among disbelievers (vs. believers). Third, based on some recent evidence linking an intuitive cognitive style (low ACS) to disgust sensitivity and endorsement of the binding moral foundations [55], it was proposed that high ACS may also contribute to lower endorsement of the binding moral foundations among disbelievers (vs. believers).

Previous research on the moral psychological consequences of analytic thinking [57, 60–63] also led to the prediction that ACS should account for disbelievers’ (vs. believers) higher inclination to rely on consequentialist moral reasoning. Finally, and more speculatively, it was proposed that ACS may also help account for disbelievers’ stronger inclination to moralize epistemic rationality.

The present research provided support for all of these predictions but one. First, results supported the predictions that CREDs, existential threat, and cognitive style should help explain differences between believers and disbelievers in their level of endorsement of the binding moral foundations. Low (vs. high) exposure to CREDs (Studies 2 & 3), low (vs. high) existential threat (Study 2), and high (vs. low) ACS (Study 3) in large part accounted for the weaker endorsement of the binding moral foundations observed among disbelievers (vs. believers) in both countries. Results also provided some support for the prediction that differences in cognitive style help explain why disbelievers (vs believers) are more inclined to rely on consequentialist moral reasoning (Study 3). Specifically, high (vs. low) ACS contributed slightly to disbelievers’ (vs. believers’) stronger reliance on consequentialist moral reasoning. However, no support was found for the prediction that differences in cognitive style contribute to differences between disbelievers and believers in their inclination to moralize epistemic rationality, as disbelief (Studies 2–3) and ACS (Study 3) explained only a miniscule amount of variance in moralized rationality.

### Theoretical and practical implications

The present findings paint a clear and consistent picture of how disbelievers in the U.S. and in Sweden conceptualize morality, and provide some initial evidence of the processes that may explain why they view morality in this way. First and foremost, this data suggests that the cross-cultural stereotype of atheists as lacking a moral compass is inaccurate. In fact, the data suggests that disbelievers share many of the moral values endorsed by believers, and they score equally low on amoral tendencies. Notably, these findings are consistent with, and complement, experience sampling research showing that disbelievers and believers engage in (self-defined) moral and immoral behaviors at the same rate [13]. At the same time, the present research also suggests that disbelievers have a more constrained view of morality than believers do, in that they are less inclined to endorse the binding moral foundations, and more inclined to judge the morality of actions that inflict harm on a consequentialist, case-by-case basis. It is worth noting that moral character evaluations of people who make decisions based on consequentialist (vs. rule-based) principles are more negative, because consequentialists are perceived as less empathic [78]. In the light of such findings it seems plausible that atheists’ inclination to rely on consequentialist principles, along with their weak endorsement of the binding moral foundations, may to some degree have contributed to their reputation as lacking in moral character.

The present findings also have implications for our understanding of where disbelievers moral values and principles stem from. Atheism merely implies the absence of religious belief, and says nothing about what *positive* beliefs the disbeliever holds. I therefore argue that disbelief itself should contribute little to the endorsement of moral values and principles. Disbelief may contribute to the *rejection* of specific moral rules that stem from, or are closely associated with, religion (e.g., “You should not eat pork”, “you should not work on the Sabbath”). However, it seems implausible that disbelief itself causes people to adopt certain moral principles (e.g., consequentialism), or to discard broad classes of values as irrelevant for morality (e.g., the binding moral foundations). Instead, it has been argued in the present article that unique features of disbelievers’ morality may stem from the cultural, (de)motivational, and cognitive antecedents of their disbelief. The present studies provided support for this line of reasoning, as three of the four presumed antecedents of disbelief examined were associated with the moral profile of disbelievers. Low exposure to CREDs, low levels of existential threat, and high ACS were associated with the weaker endorsement of the binding moral foundations observed among disbelievers (vs. believers). Furthermore, high ACS, and low exposure to CREDs were (slightly) associated with the higher levels of consequentialist thinking observed among disbelievers (vs. believers). Having said that, however, it is important to note that the present data was correlational in nature. As a consequence, conclusions cannot be drawn about the *causal* role of these presumed antecedents of disbelief in the process of moral value acquisition and rejection. I will return to this point below.

### Limitations and suggestions for future research

The present research constitutes the first systematic examination of the moral values and principles endorsed by disbelievers, and the processes through which they may be acquired. As such it represents an important first step in the process of explaining the relationship between disbelief and morality. However, the studies reported in this article have a number of limitations that should be addressed in future studies. First and foremost, the present studies relied exclusively on self-reported moral values and principles. Although this approach provided important insights regarding how disbelievers (vs. believers) think about morality, future studies should examine what the behavioral consequences are of these similarities and differences in moral values and principles. Because moral stances are often particularly strong predictors of behavior, there are reasons to believe that the differences in moral values and principles between disbelievers and believers observed in the present studies could have important behavioral implications in various domains. However, it should also be noted that religious belief is positively associated with reputational concerns [23], which can also promote prosocial behavior [24, 25]. It therefore seems plausible that moral values and principles may serve as somewhat weaker predictors of behavior among disbelievers (vs. believers), at least when other people are present [11, see also 79].

Another limitation of the present research is the exclusive reliance on cross-sectional data. Although the results are *consistent* with the notion that presumed antecedents of disbelief help explain why disbelievers’ (vs. believers’) views of morality differ, the cross-sectional designs employed in these studies do not allow for conclusions about causal relationships. Future studies should further examine these relationships using methods that enable causal conclusions. In particular, confidence in the current interpretation of these results would increase considerably with corroborating evidence from longitudinal and experimental studies.

The main purpose of the present research was to examine how people who do not believe in God think about morality, and to compare their views about morality with those of people who do believe in God. Participants in the two large cross-national surveys were therefore explicitly asked whether they believed in God or not, and those who reported not knowing whether they were believers or not were dropped. Although this classification may seem simplistic, it served to ensure that comparisons were made between people who did (vs. did not) believe in God. These studies also included a continuous measure of belief (vs. disbelief) strength. However, because scores on this measure deviated drastically from normality, and because results did not substantially alter any of the results reported in this article, those analyses were reported elsewhere (see S1 Text). It is also worth noting that results were strikingly similar when using a continuous measure of level of religiosity (Studies 1A-1B). The consistent results across different measures indicate that the present findings are not an artefact of a particular method of assessing religious disbelief (vs. belief). That said, however, future studies should expand on the present studies and examine the extent to which differences between disbelievers’ and believers’ views about morality vary as a function of the kind of religious beliefs that people endorse.

Related to the point above, although it is a notable strength of the present research that similar results were obtained across multiple studies, using large samples from two countries in which the populations differ dramatically in disbelief (vs. belief), the U.S. and Sweden are nonetheless relatively similar countries in many respects. For example, religious believers are predominantly Christian in both countries, and both cultures are WEIRD (i.e., Western, Educated, Industrialized, Rich, Democratic). Future research is needed to determine whether the moral psychological similarities and differences observed between disbelievers and believers hold up in non-WEIRD cultures, and in cultures where other religious beliefs are the norm.

## Conclusion

The purpose of the present research was to systematically examine how conceptualizations of morality differ between disbelievers and believers, and to explore whether moral psychological differences between these groups could be due to four presumed antecedents of disbelief. The results consistently indicate that disbelievers and believers, in the U.S. as well as in Sweden, are equally inclined to view the individualizing moral foundations, Liberty/oppression, and epistemic rationality as important moral values. However, these studies also point to some consistent cross-national differences in the moral psychology of disbelievers as compared to believers. Specifically, disbelievers are less inclined than believers to endorse the binding moral foundations, and more inclined to engage in consequentialist moral reasoning. The present results further suggest that these differences may stem from disparities in exposure to CREDs, levels of perceived existential threat, and individual differences in cognitive style. It seems plausible that the more constrained and consequentialist view of morality that is associated with disbelief may have contributed to the widespread reputation of atheists as immoral in nature.

## Supporting information

S1 Text Supporting analyses. (DOCX)

S2 Text List of all variables in each dataset. (DOCX)

S1 File Study 1A dataset. (SAV)

S2 File Study 1B dataset. (SAV)

S3 File Study 2 dataset. (SAV)

S4 File Study 3 dataset. (SAV)
