# Supplementary material for: The amoral atheist? A cross-national examination of cultural, motivational, and cognitive antecedents of disbelief, and their implications for morality
Source: PLoS One. 2021 Feb 24;16(2):e0246593. doi: 10.1371/journal.pone.0246593 (PMC7904147; doi:10.1371/journal.pone.0246593)
Supplement: S1 Text — (DOCX) [file pone.0246593.s001.docx]

**S1 Text. Supporting analyses**

**List of contents**

1. Main analyses: Studies 1A and 1B (Tables A-D)

2. Main analyses: Study 2 (Tables E-H)

3. Main analyses: Study 3 (Tables I-L)

4. Alternative analyses: All studies. How religiosity, belief in God, and the presumed antecedents of disbelief relate to separate moral foundations (Tables M-R)

5. Alternative analyses: Study 2. Using a continuous measure of (dis)belief strength, rather than the dichotomous belief (vs. disbelief) measure.

6. Alternative analyses: Study 2. Analyses including gender, age, and education as control variables. I did not report these analyses in the article because of drastic data loss (many participants failed to report either their age, gender, or level of education).

7. Alternative analyses: Study 3. Using including a continuous measure of (dis)belief strength, rather than the dichotomous belief (vs, disbelief) measure.

**1. Main analyses: Studies 1A and 1B (Tables A-D)**

Table A

*Means, Standard deviations, Cronbach’s Alphas, and zero-order correlations (Study 1A)*

Variable *M* *SD* α 1 2 3 4 5 6

1 Religiosity 39.28 37.07 - -

2 Political orientation 3.26 1.74 - .38* -

3 Individualizing 4.70 .71 .80 .07 -.10 -

4 Binding 3.46 .99 .92 .62* .45* .04 -

5 Liberty/Oppression 4.40 .70 .74 -.02 .17* .37* .12 -

6 Amorality 1.90 .87 .88 -.12 -.18* -.45* .02 -.16* -

* *p* < .05

Table B

*Means, Standard deviations, Cronbach’s Alphas, and zero-order correlations (Study 1B)*

Variable M SD α 1 2 3 4 5 6 7 8

1 Religiosity 32.95 35.86 - -

2 Political orientation 3.22 1.64 - .37* -

3 Individualizing 4.72 .73 .80 .04 -.23* -

4 Binding 3.38 .94 .91 .53* .48* .13* -

5 Liberty/Oppression 4.45 .73 .74 -.05 .19* .43* .18* -

6 Amorality 1.85 .89 .88 -.18* .01 -.43* -.07 -.13* -

7 SDE 5.30 3.91 .79 .10 .08 .24* .12 .28* -.21* -

8 IM 6.44 4.53 .84 .26* .11 .39* .23* .14* -.61* .50* -

* *p* < .05

Table C

*Results of hierarchical regression analyses predicting morally relevant variables (Study 1A)*

DV Predictor *b* *SE* Δ*R*^2^

Binding

Controls .07

Religiosity .016*** .002 .321

Individ.

Controls .036

Religiosity .001 .002 .003

Liberty

Controls .03

Religiosity .001 .002 .001

Amorality

Controls .207

Religiosity -.002 .002 .005

*** *p* < .001

Table D

*Results of hierarchical regression analyses predicting morally relevant variables (Study 1B)*

DV Predictor *b* *SE* Δ*R*^2^

Binding

Controls .029

Step .041

IM .045** .015

SDE -.004 .018

Religiosity .013*** .002 .224

Individ.

Controls .085

Step .115

IM .051*** .11

SDE .012 .013

Religiosity -.002* .001 .013

Liberty

Controls .041

Step .057

IM -.002 .011

SDE .047** .014

Religiosity -.002 .001 .009

Amorality

Controls .096

Step .312

IM -.119*** .011

SDE .018 .013

Religiosity .001 .001 .001

*** *p* < .001, ** *p* < .01, * *p* = .05

**2. Main analyses: Study 2 (Tables E-H)**

Table E

*Means, Standard deviations, Cronbach’s Alphas, and zero-order correlations (Study 2)*

Variable M SD α 1 2 3 4 5 6 7 8

1 Belief (vs. disbelief) .57 0.50 - -

2 Political orientation 3.78 1.68 - .22** -

3 CREDs 3.42 1.66 .96 .49** .14** -

4 BDW 4.26 1.08 .84 .19** .22** .04 -

5 Individualizing 4.53 .76 .82 .10** -.20** .11** .03 -

6 Binding 3.83 .88 .89 .43** .32** .46** .32** .41** -

7 Liberty/oppression 4.268 .74 .71 .14** .17** .20** .17** .51** .55** -

8 Moralized Rationality 4.19 .95 .74 -.09** -.02 .06** -.07** .18** .15** .19** -

* *p* < .01, ** *p* < .001

Table F

*Results of hierarchical logistic regression analyses predicting (dis)belief (Study 2)*

DV Predictor *b* *SE* *OR*

Belief (vs. disbelief) Country -1.54*** .09 .21

CREDs 1.07*** .06 2.92

BDW .45*** .05 1.57

Country by CREDs -.04 .13 .96

Country by BDW .50*** .11 1.65

*** *p* < .001

Table G

*Results of hierarchical regression analyses predicting endorsement of moral values (Study 2)*

DV Predictor *b* *SE* Δ*R*^2^

Binding

Country .483*** .037 .076

Belief .674*** .037 .125

Country x belief .416*** .074 .012

Individ.

Country .154*** .033 .01

Belief .105** .035 .004

Country x belief .02 .071 .000

Liberty

Country .277*** .031 .035

Belief .128*** .034 .006

Country x belief .139* .068 .002

Moralized Rationality

Country -.112** .041 .003

Belief -.145** .045 .005

Country by belief .044 .089 .000

*** *p* < .001, ** *p* < .01, * *p* < .05

Table H

*Results of hierarchical regression analyses predicting endorsement of binding foundations (Study 2)*

DV Predictor *b* *SE* Δ*R*^2^

Binding Country .483*** .037 .075

Step .229

CREDs .368*** .018

BDW .260*** .016

Belief .361*** .038 .029

Step .002

Country by CREDs .077* .035

Country by BDW .040 .032

*** *p* < .001, * *p* < .05

**3. Main analyses: Study 3 (Tables I-L)**

Table I

*Means, Standard deviations, Cronbach’s Alphas, and zero-order correlations (Study 3)*

Variable M SD α 1 2 3 4 5 6 7 8 9

1 Belief (vs. disbelief) .51 .50 - -

2 Political orientation 3.90 1.76 - .15** -

3 CREDs 3.75 1.60 .94 .43** .13** -

4 ACS 1.71 1.63 .72 -.25** -.07* -.23** -

5 Mentalizing 2.84 .43 .86 .06* -.06* .09** .01 -

6 Individualizing 4.24 1.10 .89 .06* -.08** .21** .06* .45** -

7 Binding 3.68 1.09 .89 .34** .21** .49** -.24** .23** .58** -

8 Liberty/oppression 4.05 1.13 .49 .10** .08** .25** -.03 .30** .68** .61** -

9 Moralized Rationality 4.26 .96 .72 -.07* .03 .18** -.03 .10** .25** .29** .32** -

10 CTS 1.56 .40 .87 -.18** -.07* -.05* .07* -.12** -.05* -.13** -.04 .04 -

* *p* < .05, ** p < .001

Table J

*Results of hierarchical logistic regression analyses predicting belief (Study 3)*

DV Predictor *b* *SE* *OR*

Belief (vs. disbelief) Gender .043 .091 1.04

Age .000 .003 1.00

Education .079** .026 1.08

Country .012 .098 1.01

CREDs 1.02*** .062 2.78

ACS -.398*** .054 .67 Mentalizing .088 .054 1.09

Country by CREDs .043 .123 1.04

Country by ACS .233* .107 1.26

Country by Mentalizing -.145 .107 .87

*** *p* < .001, ** *p* < .01, * *p* < .05

Table K

*Results of hierarchical regression analyses predicting endorsement of moral values and principles (Study 3)*

DV Predictor *b* *SE* Δ*R*^2^

Binding

Controls .017

Country .276*** .052 .014

Belief .731*** .046 .111

Country x belief .253** .092 .003

Individ.

Controls .044

Country .249*** .052 .011

Belief .117* .048 .003

Country x belief .032 .097 .000

Liberty

Controls .013

Country .034 .054 .000

Belief .20*** .051 .008

Country x belief .124 .102 .001

Moralized Rationality

Controls .026

Country -.101* .046 .002

Belief -.15*** .043 .006

Country x belief .123 .086 .001

CTS

Controls .05

Country -.077*** .019 .008

Belief -.147*** .017 .035

Country x belief -.172*** .034 .012

*** *p* < .001, ** *p* < .01, * *p* < .05

Table L

*Results of hierarchical regression analyses predicting endorsement of binding foundations and consequentialist thinking (Study 3)*

DV Predictor *b* *SE* Δ*R*^2^

Binding

Controls .018

Country .265*** .053 .013

Step .265

CREDs .467*** .023

ACS -.172*** .022 Mentalizing .209*** .022

Belief .304*** .047 .015

Step .005

Country by CREDs -.085† .044

Country by ACS -.132** .042

Country by Mentalizing .082† .042

CTS

Controls .050

Country -.08*** .019 .009

Step .012

CREDs -.022* .009

ACS .021* .009

Mentalizing -.029** .009

Belief -.153*** .019 .03

Step .002

Country by CREDs -.023 .018

Country by ACS .018 .018

Country by Mentalizing .014 .017

*** *p* < .001, ** *p* < .01, * *p* < .05, † *p* < .10

**4. Alternative analyses: All studies. How religiosity, belief in God, and the presumed antecedents of disbelief relate to separate moral foundations (Tables M-R)**

Table M

*Results of hierarchical regression analyses predicting each moral foundation (Study 1A)*

DV Predictor *b* *SE* Δ*R*^2^

Care

Controls .046

Religiosity .003 .002 .019

Fairness

Controls .011

Religiosity -.002 .001 .013

Loyalty

Controls .038

Religiosity .013^***^ .002 .217

Authority

Controls .075

Religiosity .012^***^ .002 .167

Sanctity

Controls .067

Religiosity .024^***^ .002 .357

*** *p* < .001

Table N

*Results of hierarchical regression analyses predicting each moral foundation (Study 1B)*

DV Predictor *b* *SE* Δ*R*^2^

Care

Controls .077

Step .114

IM .064^***^ .013

SDE .001 .015

Religiosity -.001 .001 .001

Fairness

Controls .046

Step .036

IM .024^*^ .012

SDE .013 .014

Religiosity -.005^**^ .001 .044

Loyalty

Controls .007

Step .007

IM .014 .015

SDE .006 .018

Religiosity .008^***^ .002 .088

Authority

Controls .037

Step .030

IM .039^*^ .016

SDE -.005 .018

Religiosity .011^***^ .002 .154

Sanctity

Controls .035

Step .068

IM .084^***^ .021

SDE -.019 .025

Religiosity .022^***^ .002 .314

*** *p* < .001, ** *p* < .01, * *p* = .05

Table O

*Results of hierarchical regression analyses predicting endorsement of each moral foundation (Study 2)*

DV Predictor *b* *SE* Δ*R*^2^

Care

Country .182^***^ .037 .011

Belief .158^***^ .039 .007

Country x belief .114 .079 .001

Fairness

Country .126^***^ .034 .007

Belief .050 .037 .001

Country x belief -.073 .073 .000

Loyalty

Country .090^*^ .040 .002

Belief .509^***^ .042 .065

Country x belief .504^***^ .083 .016

Authority

Country .457^***^ .039 .062

Belief .503^***^ .040 .064

Country by belief .396^***^ .081 .010

Sanctity

Country .905^***^ .046 .156

Belief .999^***^ .045 .162

Country by belief .356^***^ .089 .005

*** *p* < .001, ** *p* < .01, * *p* < .05

Table P

*Results of hierarchical regression analyses predicting endorsement of each of the binding moral foundations (Study 2)*

DV Predictor *b* *SE* Δ*R*^2^

Loyalty Country .088^*^ .040 .002

Step .133

CREDs .323^***^ .021

BDW .169^***^ .019

Belief .247^***^ .044 .012

Step .010

Country by CREDs .202^***^ .041

Country by BDW .017 .037

Authority Country .456^***^ .039 .062

Step .172

CREDs .287^***^ .019

BDW .281^***^ .018

Belief .223^***^ .042 .010

Step .002

Country by CREDs .078^*^ .039

Country by BDW -.013 .035

Sanctity Country .904^***^ .046 .156

Step .234

CREDs .492^***^ .022

BDW .331^***^ .019

Belief .608^***^ .045 .049

Step .003

Country by CREDs -.049 .041

Country by BDW .114^**^ .038

*** *p* < .001, ** *p* < .01, * *p* < .05

Table Q

*Results of hierarchical regression analyses predicting endorsement of each moral foundation (Study 3)*

DV Predictor *b* *SE* Δ*R*^2^

Care

Controls .039

Country .297^***^ .054 .015

Belief .108^*^  .051 .002

Country x belief .082 .102 .000

Fairness

Controls .030

Country .266^***^  .053 .012

Belief .116^*^ .050 .003

Country x belief .014 .101 .000

Loyalty

Controls .014

Country .237^***^ .055 .009

Belief .540^***^  .050 .054

Country x belief .177 .101 .001

Authority

Controls .011

Country .183^**^ .053 .006

Belief .536^***^  .049 .056

Country x belief .273^**^ .098 .004

Sanctity

Controls .020

Country .376^***^  .062 .018

Belief 1.122^***^ .052 .182

Country x belief .326^**^  .105 .004

*** *p* < .001, ** *p* < .01, * *p* < .05

Table R

*Results of hierarchical regression analyses predicting endorsement of each of the binding moral foundations (Study 3)*

DV Predictor *b* *SE* Δ*R*^2^

Loyalty

Controls .014

Country .226^***^ .055 .008

Step .195

CREDs .381^***^ .025

ACS -.148^***^ .024 Mentalizing .267^***^ .024

Belief .156^**^ .052 .004

Step .004

Country by CREDs -.090 .049

Country by ACS -.142^**^ .048

Country by Mentalizing .015 .047

Authority

Controls .011

Country .173^**^ .054 .005

Step .200

CREDs .400^***^ .024

ACS -.131^***^ .023

Mentalizing .240^***^ .023

Belief .145^***^ .051 .003

Step .007

Country by CREDs -.047 .048

Country by ACS -.182^***^ .046

Country by Mentalizing .059 .046

Sanctity

Controls .021

Country .365^***^ .062 .017

Step .283

CREDs .616^***^ .027

ACS -.236^***^ .025

Mentalizing .127^***^ .025

Belief .615^***^ .054 .043

Step .005

Country by CREDs -.117^*^  .050

Country by ACS -.069 .049

Country by Mentalizing .155^**^  .048

*** *p* < .001, ** *p* < .01, * *p* < .05, + *p* < .10

**5. Alternative analyses: Study 2. Using a continuous measure of (dis)belief strength, rather than the dichotomous belief (vs. disbelief) measure**

**Description of the (dis)belief strength measure**. Participants were asked “Do you believe that there is a God?” (Yes, No, Don’t know). Those who answered “Don’t know”, or failed to answer this question, were excluded from further analyses. Those who answered Yes (vs. No) were redirected to the appropriate version of the follow-up question “How confident are you that there is a God (vs. is no God)?” (1 = *Not at all confident*, 7 = *Extremely confident*). Responses to the disbelief strength version of this question were re-coded (-1 = *Not at all confident*, -7 = *Extremely confident*), and then merged with responses to the belief strength version to create a 14-point (dis)belief strength scale (-7 = *Extremely confident that there is no God*, 7 = *Extremely confident that there is a God*).

**Predicting (dis)belief strength.** As should expected, given the higher number of believers in the U.S. (vs. Swedish) sample, there was a substantial difference in (dis)belief strength between Americans (*M* = 3.60, *SD* = 6.35) and Swedes (*M* = -1.85, *SD* = 6.78), *t*(2128) = 19.13, *p* < .001, *d* = 0.83. We conducted a hierarchical regression analysis, using the continuous measure of (dis)belief strength as the criterion. Country of residence (1 = U.S., 0 = SWE) was entered in Step 1, and the presumed antecedents of (dis)belief (CREDs, BDW) were entered in Step 2. Interactions between country of residence and the presumed antecedents of (dis)belief were entered in Step 3. We limit our discussion to steps that explain at least 1% of the variance.

Consistent with the *t*-test reported above, Swedes (*b* = 5.46, *p* < .001) reported stronger disbelief than Americans, *R*^2^ = .147, *F*(1, 2118) = 366.03, *p* < .001. More importantly, the presumed antecedents of disbelief explained a large amount of additional variance, ΔR^2^ = .174, *F*(2, 2116) = 271.00, *p* < .001. As predicted, less exposure to CREDs (*b* = 3.01, *p* < .001), and lower BDW (*b* = 1.19, *p* < .001) was associated with stronger disbelief. There were only very minor cultural differences in predictors of (dis)belief strength, although both the country by CREDs (*b* = -.73, *p* < .001) and the country by BDW (*b* = 1.24, *p* < .001) interactions were significant. Exposure to CREDs was a slightly stronger predictor of (dis)belief strength in Sweden (*b* = 3.37, *p* < .001) than in the U.S. (*b* = 2.02, *p* < .001), whereas BDW was a stronger predictor of (dis)belief strength in the U.S. (*b* = 1.89, *p* < .001) than in Sweden (*b* = .65, *p* < .001).

(**Dis)belief strength and moral values**. We examined the associations between (dis)belief and the moral values in a set of hierarchical regression analyses. Country of residence (1 = U.S., 0 = SWE) was entered in Step 1. We then entered (dis)belief strength in Step 2. Finally, we entered the interaction term between country and the continuous (dis)belief strength measure in Step 3.

*Binding moral foundations*. Americans (*b* = .483, *p* < .001) endorsed the binding moral foundations more than Swedes, *R*^2^ = .076, *F*(1, 2112) = 172.53, *p* < .001. More importantly, and consistent with Studies 1A and 1B, stronger disbelief (*b* = .328, *p* < .001) was associated with weaker endorsement of the binding moral foundations, Δ*R*^2^ = .14, *F*(1, 2111) = 376.96, *p* < .001. The country by (dis)belief strength interaction (*b* = .188, *p* < .001) only accounted for a small amount of variance, Δ*R*^2^ = .012, *F*(1, 2110) = 31.43, *p* < .001. Stronger disbelief was somewhat more closely associated with endorsement of the binding moral foundations in the U.S. (*b* = .426, *p* < .001) than in Sweden (*b* = .238, *p* < .001).

*Individualizing moral foundations*. Americans endorsed the individualizing foundations slightly more than Swedes (*b* = .154, *p* < .001), Δ*R*^2^ = .01, *F*(1, 2116) = 22.29, *p* < .001. However, neither (dis)belief strength, Δ*R*^2^ = .005, *F*(1, 2115) = 10.49, *p* = .001, nor the country by (dis)belief interaction explained any substantial amount of variance, Δ*R*^2^ = .000, *F*(1, 2114) = .06, *p* = .82.

*Liberty/oppression*. Americans (*b* = .278, *p* < .001) endorsed Liberty/oppression to a larger extent than Swedes, Δ*R*^2^ = .036, *F*(1, 2117) = 77.96, *p* < .001. More importantly, and consistent with Studies 1A and 1B, (dis)belief strength (*b* = .067, *p* < .001) did not account for any substantial variance in endorsement of Liberty/oppression, Δ*R*^2^ = .008, *F*(1, 2116) = 18.18, *p* < .001; neither did the country by (dis)belief strength interaction, Δ*R*^2^ = .002, *F*(1, 2115) = 4.96, *p* < .05.

*Moralized rationality*. Country (*b* = -.112, *p* = .007) did not explain any substantial amount of variance in the inclination to moralize rationality, Δ*R*^2^ = .003, *F*(1, 2118) = 7.40, *p* = .007. More importantly, although (dis)belief was associated with slightly stronger moralization of epistemic rationality (*b* = -.066, *p* = .001), this relationship accounted for a miniscule amount of variance, Δ*R*^2^ = .005, *F*(1, 2117) = 10.54, *p* = .001, and there was no country by (dis)belief strength interaction, Δ*R*^2^ = .000, *F*(1, 2116) = .28, *p* = .60.

**Can theorized** **antecedents of (dis)belief explain why disbelievers are less inclined than believers to endorse the binding moral foundations?** To address this question, we once again conducted hierarchical regression analyses. Country of residence was entered in Step 1, followed by the antecedents of disbelief (CREDs, BDW) in Step 2. (Dis)belief strength was entered in Step 3, to examine its contributions to endorsement of the binding foundations once the variance explained by the antecedents of (dis)belief strength had been accounted for. Finally, we entered the interaction terms between country of residence and the two presumed antecedents of (dis)belief strength in Step 4.

The theorized antecedents of (dis)belief explained a large amount of variance in endorsement of binding values, Δ*R*^2^ = .229, *F*(2, 2101) = 346.81, *p* < .001. As hypothesized, less exposure to CREDs (*b* = .368, *p* < .001), and lower BDW (*b* = .26, *p* < .001), were associated with considerably weaker endorsement of binding moral values. Once these associations had been accounted for, the link between (dis)belief strength and endorsement of binding moral values was weakened considerably, Δ*R*^2^ = .034, *F*(1, 2100) = 107.14, *p* < .001. Moreover, these patterns were similar in both countries, as the country by CREDs and country by BDW interactions failed to account for any substantial amount of variance. Taken together, these results are consistent with the notion that less exposure to CREDs and lower BDW help explain why disbelievers (vs. believers) are less inclined to endorse the binding moral foundations.

**6. Alternative analyses: Study 2. Analyses including gender, age, and education as control variables**

**Predicting (dis)belief.** We conducted a hierarchical logistic regression using the dichotomous disbelief measure as our criterion (1 = Believe there is a God, 0 = Believe there is no God). We entered gender, age, and level of education in Step 1. Country of residence (1 = U.S., 0 = SWE) was added in Step 2, and the presumed antecedents of (dis)belief (CREDs, BDW) were entered in Step 3. Interactions between country of residence and the presumed antecedents of (dis)belief were entered in Step 4.

Less exposure to CREDs was associated with a higher probability of being a disbeliever, (*b* = 1.12, SE = .08, χ^2^(1) = 200.05, *p* < .001, odds ratio = 3.06), as were lower levels of BDW (*b* = .51, SE = .07, χ^2^(1) = 52.26, *p* < .001, odds ratio = 1.66).

(**Dis)belief and moral values**. We entered gender, age, and level of education in Step 1. Country of residence (1 = U.S., 0 = SWE) was entered in Step 2. We then entered (dis)belief in Step 3, Finally, we added the interaction term between country and (dis)belief in Step 4.

*Binding moral foundations*. Whereas the control variables did not explain any variance in the binding moral foundations, *R*^2^ = .003, *F*(3, 1424) = 1.28, *p* = .28, country of residence did, Δ*R*^2^ = .072, *F*(1, 1423) = 111.34, *p* < .001. Americans endorsed the binding moral foundations more than Swedes (*b* = .492, *p* < .001). More importantly, (dis)belief explained a considerable amount of additional variance, Δ*R*^2^ = .147, *F*(1, 1422) = 267.94, *p* < .001, whereas the interaction was miniscule in size, Δ*R*^2^ = .009, *F*(1, 1421) = 16.62, *p* < .001.

*Individualizing moral foundations*. Being female (*b* = .322, *p* < .001), and of older age (*b* = .006, *p* < .001) was associated with stronger value endorsement, *R*^2^ = .056, *F*(3, 1425) = 28.38, *p* < .001. Country of residence, Δ*R*^2^ = .007, *F*(1, 1424) = 10.82, *p* = .001, (Dis)belief (Δ*R*^2^ = .002), and the country by (dis)belief interaction (Δ*R*^2^ = .000), were virtually unrelated to endorsement of the individualizing moral foundations.

*Liberty/oppression*. Control variables did not explain any variance in endorsement of Liberty/oppression, *R*^2^ = .003, *F*(3, 1427) = 1.34, *p* = .26. However, Americans (*b* = .295, *p* < .001) endorsed Liberty/oppression to a larger extent than Swedes, Δ*R*^2^ = .040, *F*(1, 1426) = 59.07, *p* < .001. (Dis)belief (*b* = .151, *p* < .001) explained only a miniscule amount of variance in endorsement of Liberty/oppression, Δ*R*^2^ = .009, *F*(1, 1425) = 14.05, *p* < .001, as did the interaction, Δ*R*^2^ = .003, *F*(1, 1424) = 3.89, *p* < .05.

*Moralized rationality*. Being male (*b* = -.358, *p* < .001), and younger (*b* = -.008, *p* < .001), was associated with stronger endorsement of epistemic rationality as an important moral value, *R*^2^ = .046, *F*(3, 1430) = 22.82, *p* < .001. Swedes (*b* = -.199, *p* < .001) were also somewhat more inclined than Americans to moralize rationality, Δ*R*^2^ = .01, *F*(1, 1429) = 15.14, *p* < .001. More importantly, (dis)belief (Δ*R*^2^ = .003), and the country by (dis)belief interaction (Δ*R*^2^ = .000). explained miniscule amounts of variance in moralized rationality.

**Can theorized** **antecedents of (dis)belief explain why disbelievers are less inclined than believers to endorse binding moral values?** We once again conducted a hierarchical regression analysis. Control variables (gender, age, education) were entered in Step 1, followed by country of residence in Step 2. We then entered the antecedents of disbelief (CREDs, BDW) in Step 3. (Dis)belief was entered in Step 4, to examine its contributions to morality once the variance explained by the antecedents of (dis)belief had been accounted for. Finally, we entered the interaction terms between country of residence and the two presumed antecedents of (dis)belief in Step 5.

The theorized antecedents of (dis)belief explained a large amount of variance in endorsement of binding values, Δ*R*^2^ = .249, *F*(2, 1416) = 260.23, *p* < .001. As hypothesized, less exposure to CREDs (*b* = .392, *p* < .001), and lower BDW (*b* = .254, *p* < .001), were associated with considerably weaker endorsement of binding moral values. Once these associations had been accounted for, the link between (dis)belief and endorsement of binding moral values was weakened considerably, Δ*R*^2^ = .035, *F*(1, 1415) = 76.48, *p* < .001.

**7. Alternative analyses: Study 3. Using including a continuous measure of (dis)belief strength, rather than the dichotomous belief (vs, disbelief) measure**

As in Study 2, we examined the relationships between the presumed antecedents of (dis)belief and reported (dis)belief strength in using hierarchical linear regression. Control variables (gender, age, education) were entered in Step 1, followed by country of residence in Step 2. In Step 3 we entered the three presumed antecedents of (dis)belief (CREDs, ACS, Mentalizing), followed by their interaction terms with country of residence in Step 4.

Neither the control variables (*R*^2^ = .007), nor country of residence (Δ*R*^2^ = .001) accounted for much variance in (dis)belief strength. However, the presumed antecedents of (dis)belief did explain a considerable amount of variance, ΔR^2^ = .236, *F*(1, 1962) = 204.11, *p* < .001. As predicted, less exposure to CREDs in the community was associated with stronger disbelief (*b* = 2.68, *p* < .001), as was a more analytic cognitive style (*b* = -.977, *p* < .001). By contrast, mentalizing abilities were not reliably associated with (dis)belief strength (*b* = .186, *p* = .13), and none of the interaction terms explained any variance in (dis)belief strength, ΔR^2^ = .001, *F*(1, 1959) = 1.25, *p* = .29.

**(Dis)belief strength and morality**. As in Study 2, we examined the links between (dis)belief and moral values (and principles) in a set of hierarchical regression analyses. The analyses were carried out the exact same way as in Study 2.

*Binding moral foundations*. The control variables explained some variance, *R*^2^ = .017, *F*(3, 1980) = 1.74, *p* < .001, as a higher level of education was associated with stronger endorsement of the binding foundations (*b* = .078, *p* < .001). Step 2 explained additional variance, Δ*R*^2^ = .013, *F*(1, 1979) = 26.34, *p* < .001, as Americans endorsed the binding foundations somewhat more than Swedes (*b* = .265, *p* < .001). More importantly, entering (dis)belief strength in Step 3 accounted for considerable additional variance, Δ*R*^2^ = .13, *F*(1, 1978) = 297.95, *p* < .001. As in the previous studies, stronger disbelief was associated with weaker endorsement of binding values (*b* = .39, *p* < .001). Furthermore, we found no cultural differences in this pattern, as adding the country by disbelief strength interaction term explained very little variance, Δ*R*^2^ = .002, *F*(1, 1977) = 4.11, *p* = .04.

*Individualizing moral foundations*. Control variables accounted for some variance, *R*^2^ = .037, *F*(3, 1984) = 25.69, *p* < .001. Women (*b* = .268, *p* < .001), older (*b* = .006, *p* < .001), and more educated participants (*b* = .065, *p* < .001) were all more inclined to endorse the individualizing moral foundations. Country also explained some variance, Δ*R*^2^ = .014, *F*(1, 1983) = 29.85, *p* < .001, as Americans (vs. Swedes) were somewhat more inclined to endorse the individualizing moral foundations (*b* = .28, *p* < .001). More importantly, however, (dis)belief strength only accounted for a miniscule amount of variance, Δ*R*^2^ = .003, *F*(1, 1982) = 6.89, *p* = .009, and the country by (dis)belief strength interaction was not significant, Δ*R*^2^ = .000, *F*(1, 1981) < 1, *p* = .94.

*Moralized rationality*. Control variables accounted for some variance, *R*^2^ = .025, *F*(3, 1982) = 25.69, *p* < .001. Being male (*b* = -.22, *p* < .001), and more educated (*b* = .047, *p* < .001) was associated with stronger endorsement of epistemic rationality as a moral value. None of the subsequent steps accounted for a substantial amount of variance (Δ*R*^2^ < .007%). *Consequentialism*. Control variables accounted for variance in the CTS, *R*^2^ = .049, *F*(3, 1970) = 33.68, *p* < .001, as being male (*b* = -.087, *p* < .001), younger (*b* = -.004, *p* < .001), and more educated (*b* = .018, *p* < .001) were associated with higher scores on consequentialism. Country did not explain a substantial amount of variance, Δ*R*^2^ = .008, *F*(1, 1969) = 17.34, *p* < .001. However, (dis)belief strength did explain additional variance, Δ*R*^2^ = .036, *F*(1, 1968) = 77.86, *p* < .001, as stronger disbelief was associated with a more consequentialist view of morality (*b* = -.072, *p* < .001). Finally, there was also a small but reliable interaction effect (*b* = -.09, *p* < .001) between country and (dis)belief strength, Δ*R*^2^ = .013, *F*(1, 1967) = 27.62, *p* < .001. Stronger disbelief was associated with a more consequentialist view of morality among Americans *(b* = -.116, *p* < .001), whereas this association was not reliable among Swedes (*b* = -.023, *p* = .09).

**Can theorized** **antecedents of (dis)belief explain differences in morality between believers and disbelievers?** We restricted our analyses to the binding moral foundations, and consequentialist thinking, as these moral considerations were the only ones that were predicted by (dis)belief strength. Control variables (gender, age, education) were entered in Step 1, followed by country of residence in Step 2. We then entered the antecedents of disbelief (CREDs, ACS, Mentalizing abilities) in Step 3. (Dis)belief strength was entered in Step 4, to examine its contributions to morality once the variance explained by the presumed antecedents of (dis)belief strength had been accounted for. Finally, we entered the interaction terms between country of residence and the three presumed antecedents of (un)belief strength in Step 5.

*Binding values*. As predicted, less exposure to CREDs (*b* = .467, *p* < .001), and a more analytic cognitive style (*b* = -.172, *p* < .001) were associated with weaker endorsement of binding values. Lower mentalizing abilities were also associated with weaker endorsement of the binding foundations (*b* = .21, *p* < .001), and these three factors accounted for a considerable amount of variance, ΔR^2^ = .266, *F*(3, 1954) = 246.04, *p* < .001. Moreover, once the associations to these factors had been accounted for, (dis)belief strength only accounted for a very small amount of variance in endorsement of binding values, ΔR^2^ = .019, *F*(1, 1953) = 53.30, *p* < .001, as did the interaction terms, ΔR^2^ = .006, *F*(2, 1951) = 5.23, *p* = .003. Thus, the results are consistent with the notion that the links between (dis)belief strength and endorsement of binding moral values are predominantly due to underlying differences between believers and disbelievers in their level of exposure to CREDs, and in analytic cognitive style. We further note that lower mentalizing abilities were associated with weaker endorsement of binding values, but that this relationship cannot account for differences in endorsement of binding values between believers and disbelievers, as mentalizing abilities were unrelated to (dis)belief strength in this study.

*Consequentialist thinking*. As predicted, a more analytic cognitive style (*b* = .022, *p* < .05), lower mentalizing abilities (*b* = -.03, *p* = .001), and less exposure to CREDs (*b* = -.02, *p* < .05) were associated with more consequentialist thinking. It should be noted, however, that these variables only accounted for a modest amount of variance, Δ*R*^2^ = .013, *F*(3, 1941) = 8.82, *p* < .001. Furthermore, (dis)belief strength remained a stronger predictor of consequentialist thinking, Δ*R*^2^ = .031, *F*(1, 1940) = 67.58, *p* < .001. The interaction terms did not predict a significant amount of variance in consequentialist thinking, ΔR^2^ = .002, *F*(3, 1937) = 1.14, *p* < .33. These results suggest that, although the presumed antecedents of (dis)belief can account for a small portion of the association between (dis)belief and consequentialist thinking, there appear to be additional factors that contribute to more consequentialist thinking among disbelievers (vs. believers).
