# Supplementary material for: The amoral atheist? A cross-national examination of cultural, motivational, and cognitive antecedents of disbelief, and their implications for morality
Source: PLoS One. 2021 Feb 24;16(2):e0246593. doi: 10.1371/journal.pone.0246593 (PMC7904147; doi:10.1371/journal.pone.0246593)
Supplement: S2 Text — (DOCX) [file pone.0246593.s002.docx]

**S2 Text**

**Study 1A Variable list**

Religiosity = Level of (ir)religiosity

Relpref = Religious preference

Jewish_den = Jewish denominations

Chr_den = Christian denominations

Other_rel = Other religious denominations

Non_rel = Nonreligious identity

Amorality 1-5 = Amoral tendencies

Harm 1-6 = Care/Harm foundation (MFQ)

Fairness 1-6 = Fairness/cheating foundation (MFQ)

Loyalty 1-6 = Loyalty/betrayal foundation (MFQ)

Authority 1-6 = Authority/subversion foundation (MFQ)

Purity 1-6 = Sanctity/degradation foundation (MFQ)

Liberty 1-9 = Liberty/oppression foundation

Gender = Gender of participant

Age = Age of participant

Race = Race/ethnicity of participant

Education = Level of education

PO = Political orientation

AmoralityScale = Scale based on average from Amorality 1-5

IndividualizingScale = Average score based on Harm 1-6, and Fairness 1-6

BindingScale = Average score based on Loyalty 1-6, Authority 1-6, and Purity 1-6

LibertyScale = Average score based on Liberty 1-9

HarmScale = Average score based on Harm 1-6

FairnessScale = Average score based on Fairness 1-6

LoyaltyScale = Average score based on Loyalty 1-6

AuthorityScale = Average score based on Authority 1-6

SanctityScale = Average score based on Purity 1-6

**Study 1B Variable list**

Relpref = Religious preference

Jewish_den = Jewish denominations

Chr_den = Christian denominations

Other_rel = Other religious denominations

Non_rel = Nonreligious identity

Religiosity = Level of (ir)religiosity

DesRes 1-39 = Socially desirable responding

Amorality 1-5 = Amoral tendencies

Harm 1-6 = Care/Harm foundation (MFQ)

Fairness 1-6 = Fairness/cheating foundation (MFQ)

Loyalty 1-6 = Loyalty/betrayal foundation (MFQ)

Authority 1-6 = Authority/subversion foundation (MFQ)

Purity 1-6 = Sanctity/degradation foundation (MFQ)

Liberty 1-9 = Liberty/oppression foundation

Gender = Gender of participant

Age = Age of participant

Race = Race/ethnicity of participant

Education = Level of education

PO = Political orientation

AmoralityScale = Scale based on average from Amorality 1-5

IndividualizingScale = Average score based on Harm 1-6, and Fairness 1-6

BindingScale = Average score based on Loyalty 1-6, Authority 1-6, and Purity 1-6

LibertyScale = Average score based on Liberty 1-9

SDE 1-19 = Self-deceptive enhancement scores (From DesRes 1-39)

IM 1-20 = Impression Management (From DesRes 1-39)

SDEscale = Self-deceptive enhancement scale

IMscale = Impression management scale

HarmScale = Average score based on Harm 1-6

FairnessScale = Average score based on Fairness 1-6

LoyaltyScale = Average score based on Loyalty 1-6

AuthorityScale = Average score based on Authority 1-6

SanctityScale = Average score based on Purity 1-6

**Study 2 Variable list**

Harm 1-6 = Care/Harm foundation (MFQ)

Fair 1-6 = Fairness/cheating foundation (MFQ)

Loy 1-6 = Loyalty/betrayal foundation (MFQ)

Auth 1-6 = Authority/subversion foundation (MFQ)

Pur 1-6 = Sanctity/degradation foundation (MFQ)

Lib 1-9 = Liberty/oppression foundation

BDW 1-12 = Belief in a Dangerous World

CRED 1-7 = Exposure to credibility-enhancing displays

Belief = Do you believe that there is a God? (1 = Yes, 2 = No)

RelPref = Religious preference

Christians = Christian denominations

OtherRel = Other religious denominations

NonRel = Nonreligious identity

Gender = Gender of participant

Age = Age of participant

Race = Race/ethnicity of participant

Education = Level of education

POsocial = Political orientation on social issues

POeco = Political orientation on economic issues

MR 1-9 = Moralized Rationality

MRS = Moralized Sationality Scale

Country = Country of residence

BS = Belief Strength

Liberty = Average score based on Lib 1-9

CRED = Average score based on CRED 1-7

BDW = Average score based on BDW 1-12

Binding = Average score based on Loy 1-6, Auth 1-6, and Pur 1-6

Individ = Average score based on Harm 1-6, and Fair 1-6

ZCRED = CRED standardized

ZBDW = BDW standardized

CountrybyZCRED = Interaction term

CountrybyZBDW = Interaction term

ZBS = BS standardized

CountrybyZBS = Interaction term

BeliefDummy = Do you believe that there is a God? (1 = Yes, 0 = No)

CountrybyBeliefDummy = Interaction term

PO = Average score based on POsocial, and POeco

ZPO = PO standardized

HarmScale = Average score based on Harm 1-6

FairnessScale = Average score based on Fairness 1-6

LoyaltyScale = Average score based on Loyalty 1-6

AuthorityScale = Average score based on Authority 1-6

SanctityScale = Average score based on Purity 1-6

**Study 3 Variable list**

Country = Country of residence

Belief = Do you believe that there is a God (1 = Yes, 2 = No)

CRT 1-6 = Response to CRT problems

CRTscore 1-6 = Score on CRT problems (1 = correct, 0 = incorrect)

EQ 1-22 = Empathy Quotient

CRED 1-7 = Credibility-enhancing displays

CTS 1-14 = Consequentialist thinking

Harm 1-3 = Care/harm foundation

Fair 1-3 = Fairness/cheating foundation

Loy 1-3 = Loyalty/betrayal foundation

Aut 1-3 = Authority/subversion foundation

Pur 1-3 = Sanctity/degradation foundation

Lib 1-2 = Liberty/oppression foundation

MR 1-9 = Moralized rationality

RelAffil = Religious affiliation

Christians = Christian denominations

OtherRel = Other religious denominations

NonRel = Nonreligious identity

Gender = Gender of participant

Age = Age of participant

Race = Race/ethnicity of participant

Education = Level of education

POsocial = Political orientation on social issues

POeco = Political orientation on economic issues

EQscale = Average score based on EQ 1-22

CREDscale = Average score based on CRED 1-7

CTSscale = Average score based on CTS 1-14

Libertyscale = Average score based on Lib 1-2

Individualizing = Average score based on Harm 1-3, and Fair 1-3

Binding = Average score based on Loy 1-3, Aut 1-3, and Pur 1-3

MRS = Average score based on MR 1-9

CRTscale = Sum of CRTscore 1-6

BS = Belief strength

ZCRTscale = CRTscale standardized

ZCREDscale = CREDscale standardized

ZEQscale = EQscale standardized

CountryDummy = Country of residence (1 = US, 0 = SWE)

CountrybyZCRT = Interaction term

CountrybyZCRED = Interaction term

CountrybyZEQ = Interaction term

ZBS = BS standardized

CountrybyZBS = Interaction term

BeliefDummy = Do you believe that there is a God? (1 = Yes, 0 = No)

CountrybyBeliefDummy = Interaction term

PO = Average score based on POsocial and POeco

HarmScale = Average score based on Harm 1-3

FairnessScale = Average score based on Fairness 1-3

LoyaltyScale = Average score based on Loyalty 1-3

AuthorityScale = Average score based on Authority 1-3

SanctityScale = Average score based on Purity 1-3
